# Deep learning of dynamically responsive chemical Hamiltonians with semiempirical quantum mechanics

**DOI:** 10.1073/pnas.2120333119

**Published:** 2022-07-01

**Authors:** Guoqing Zhou, Nicholas Lubbers, Kipton Barros, Sergei Tretiak, Benjamin Nebgen

**Affiliations:** ^a^Theoretical Division, Los Alamos National Laboratory, Los Alamos, NM 87545;; ^b^Center of Nonlinear Studies, Los Alamos National Laboratory, Los Alamos, NM 87545;; ^c^Computer, Computational and Statistical Sciences Division, Los Alamos National Laboratory, Los Alamos, NM 87545;; ^d^Center for Integrated Nanotechnologies, Los Alamos National Laboratory, Los Alamos, NM 87545

**Keywords:** Hamiltonian, machine learning, semiempirical quantum chemistry, model transferability

## Abstract

Conventional machine-learning (ML) models in computational chemistry learn to directly predict molecular properties using quantum chemistry only for reference data. While these heuristic ML methods show quantum-level accuracy with speeds several orders of magnitude faster than traditional quantum chemistry methods, they suffer from poor extensibility and transferability; i.e., their accuracy degrades on large or new chemical systems. Incorporating quantum chemistry frameworks into the ML models directly solves this problem. Here we take the structure of semiempirical quantum mechanics (SEQM) methods to construct dynamically responsive Hamiltonians. SEQM methods use empirical parameters fitted to experimental properties to construct reduced-order Hamiltonians, facilitating much faster calculations than ab initio methods but with compromised accuracy. By replacing these static parameters with machine-learned dynamic values inferred from the local environment, we greatly improve the accuracy of the SEQM methods. Trained on molecular energies and atomic forces, these dynamically generated Hamiltonian parameters show a strong correlation with atomic hybridization and bonding. Trained with only about 60,000 small organic molecular conformers, the resulting model retains interpretability, extensibility, and transferability when testing on much larger chemical systems and predicting various molecular properties. Overall, this work demonstrates the virtues of incorporating physics-based descriptions with ML to develop models that are simultaneously accurate, transferable, and interpretable.

## Significance

Machine learning is revolutionizing computational chemistry by greatly reducing the computational difficulty of many simulations performed by computational chemists while maintaining accuracies of 1 kcal/mol or better. A major challenge in this field is addressing the poor extensibility and transferability of conventional machine-learning (ML) models, which result in degraded accuracy when applying these models to large or new chemical systems. To build a more general and interpretable model, we incorporate a quantum chemistry framework into the deep neural network, resulting in an interpretable Hamiltonian-based model with markedly high training efficiency. We validate this method on multiple large biochemical molecules by predicting various properties with consistently high accuracies, indicating the model is both extensible and transferable.

Modeling the interactions between electrons and nuclei is central to the study of chemical and material systems. Conventional quantum-mechanical (QM) approximations include density functional theory (DFT), coupled-cluster (CC), and configuration interaction (CI) methods (1–7). These techniques can often provide highly accurate predictions of physical properties. However, ab initio QM approaches are computationally expensive relative to alternatives such as classical force fields or semiempirical QM, which inhibit the application of these methods to very large systems and high-throughput screening of materials. Fortunately, in recent years, machine-learning (ML) methods have shown promise for making predictions with QM-level accuracy but at a much-reduced computational cost.

ML is now frequently used to make direct predictions of materials and chemistry properties. A common strategy is to extract descriptors that characterize local atomic geometries and feed them into a regression model such as a multilayer neural network (NN). NN architectures of this type include hierarchical interacting-particle neural network (HIPNN) (8), MoleculeNet (9), TensorMol (Tensorflow Molecules) (10), DPMD (Deep Potential Molecular Dynamics) (11), SchNet (12–14), ANI-1 (Accurate NeurAl Network Engine for Molecular Energies) (15–18), and PhysNet (19), etc. While these methods are predominantly used to construct potential energy surfaces and atomic forces, they have also been used for predicting various properties such as atomic charges (20, 21), dipoles (10, 22, 23), spin distributions (24), band gaps, and more (25, 26). These advances are already enabling large-scale molecular dynamics (MD) simulations with unprecedented accuracy.

Despite these successes, ML models trained to directly predict material properties lack the ability to describe properties for which a training set is not readily available. Training datasets, which typically take the form of millions of atomic configurations to cover the chemical and conformational space of interest with the desired property precomputed, are computationally very expensive to generate. This contrasts with QM approaches, which provide most desired properties in one calculation, such as energy, orbital, and charge density information. Additionally, most existing ML methods struggle to predict intensive properties of a system (i.e., properties independent of the system size), which may typically include electron delocalization effects, excited-state transition energies, etc. As such, they are often limited to specific types of systems (27–30). Typical ML models employ a nearsightedness principle, forcing certain properties, such as the energy, to be expressed as a sum over local contributions (plus long-range interactions of known form, such as Coulomb). In many cases, however, one wants to predict properties that are relevant to long-range and many-body effects and do not have a simple functional form. For example, despite efforts to predict some excited-state quantities such as nonadiabatic couplings (31, 32), true transferability in ML predictions of excited-state properties remain limited. While some work has shown that singlet–triplet gaps may be predictable in a general way (24), it is a grand challenge for these methods to be applied more generally to molecular orbital-derived properties or extend to significantly larger systems such as lipids or proteins. A final challenge with most existing ML models is interpretability and uncertainty quantification. It is difficult to understand why certain predictions are made and thus difficult to trust them.

Incorporating more physical knowledge into the ML model may help to improve transferability. One approach pioneered by Yaron and coworkers (33) uses NN and spline-based ML models to predict matrix elements for the self-consistent-charge density functional tight-binding (SCC-DFTB) Hamiltonian. This automatic parameterization technique is adapted later in the extended Hückel method and shows great interpretability (25). Another strategy leverages Δ-learning, whereby an ML model makes corrections to low-cost quantum chemistry models (34, 35) and modifies them to resemble more expensive calculations using a NN. Specifically, OrbNet uses symmetry-adapted atomic orbital features from semiempirical calculations to achieve high learning efficiency and a great reduction of computational cost (36).

Here we present a different take on the interface between ML and QM by dynamically parameterizing an effective Hamiltonian with a ML model. Established semiempirical quantum mechanics (SEQM) (37–39) methods take advantage of domain knowledge in quantum chemistry, while the HIPNN (8) facilitates dynamic alterations to the model to increase its accuracy. HIPNN behaves as an encoder, learning to predict SEQM Hamiltonian parameters from a local environment for each atom. This Hamiltonian-based method (denoted as HIPNN+SEQM), with these tuned parameters, then solves the Hartree–Fock equations for interacting electrons in a reduced-dimensional space. Thus, this method retains the structure of semiempirical QM for considering nonlocal effects through the self-consistent field (SCF) procedure and explicit Coulombic interaction terms. We have enabled back propagation through the SCF procedure, making multitask training with molecular energies, forces, orbital energies, and other properties possible. By incorporating known physics, we can achieve strong transferability and extensibility using a small amount of training data. Another advantage is that the method can naturally extend to new atom types by reusing existing SEQM parameterizations. A final advantage is interpretability: The NN modifies parameters such as “orbital energy” or the “orbital radial exponent term,” which have established physical meaning. Furthermore, we will show that the alteration of these parameters strongly correlates with traditional notions of atomic orbital hybridization and bonding in quantum chemistry, which verifies the interpretability of the model and gives insights into the electronic structure of atoms in different chemical environments.

Four models are explored in this work and applied to nonequilibrium configurations of a diverse set of organic molecules. First, for comparison, we report results for an unmodified SEQM method: Parametric Method 3 (PM3) with the D3H4 corrections on hydrogen bonding and dispersion (40–42). Second, we optimize static (constant) parameters for PM3 using ML tools, which we denote as PM3*, attempting to best fit the PM3+D3H4 form to the ab initio training data (i.e., reference DFT results). Third, we build a pure HIPNN model whose architecture was originally reported in ref. 8. HIPNN learns to directly predict energies and forces of nonequilibrium conformations. Finally, based on the PM3* parameters along with D3H4, we build the Hamiltonian-based HIPNN+SEQM model that uses HIPNN to dynamically predict PM3 parameters. The SEQM module used here is implemented in the PYSEQM software package that utilizes Pytorch to interface with other ML packages (43, 44). We chose PM3 as the base semiempirical method because it is a highly used method based on Hartree–Fock theory with well-documented features and one of the simplest methods to utilize the SCF procedure. It does not have complications like an overlap matrix or d orbitals as in other SEQM methods (45–47), which reduces the numerical instabilities and makes PM3 efficient and stable to train. To examine and compare the extensibility of these models, we benchmark all four models on the COMP6 (Comprehensive Machine-learning Potential) dataset (48), which contains diverse molecular families much larger than those found in the training set. To verify the accuracy of force predictions, we compute vibrational spectra for the Drug Bank subset of COMP6 and compare them to vibrational spectra produced by the reference method. Additionally, we study the performance of these models under a variety of nonequilibrium conditions. Finally, we examine the accuracy of the three Hamiltonian models when predicting untrained properties (which HIPNN cannot provide) to examine and compare their transferability.

## HIPNN+SEQM Workflow

**Fig. 1.**
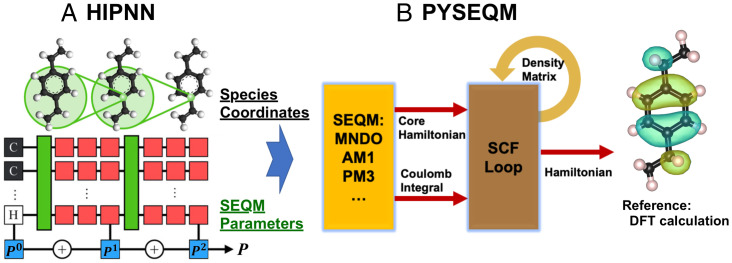
Model structure scheme. (*A*) HIPNN with molecular configurations as input (black and white blocks), interacting layers (green blocks), onsite layers (red blocks), and inference layers (blue blocks). The total correction to an SEQM parameter is obtained by summing the output from each inference layer, $P^{0},\;P^{1},\;P^{2}$. (*B*) The SEQM module takes molecule configurations and dynamic Hamiltonian parameters from HIPNN to generate core Hamiltonian and Coulomb integrals (orange block) and then performs the SCF procedure to get chemical Hamiltonians (brown block) and to predict various molecular properties. Backpropagation through the entire procedure enables stochastic gradient descent training to reference DFT data.

We illustrate the full workflow of HIPNN+SEQM here in Fig. 1. The HIPNN architecture was originally presented in ref. 8 and is briefly summarized in *SI Appendix*, section S1. HIPNN models take molecular configurations as input, where each molecule is represented as a set of atom types and pairwise interatomic distances. The input features are passed through on-site layers (red block in Fig. 1*A*), which are applied to the local features for each individual atom and shared through continuous message-passing layers (green block in Fig. 1*A*) that pass information between nearby atoms and allow atoms to see their chemical environments. An inference layer is applied to the output from each of the last on-site layers to obtain the output from the NN in sequences to get zero- to higher-order corrections of PM3 Hamiltonian parameters. Entry-wise additions are then used with the constant PM3 parameters (we use the PM3* parameter set in this work) to yield the dynamic (local environment-dependent) Hamiltonian parameters for the SEQM layers.

The Hamiltonian parameters are then fed into the SEQM module, which is detailed in the PYSEQM paper (43). PYSEQM is an open-source package for several SEQM methods, including the PM3 (40, 41) used here. They are implemented in an efficient, scalable, and stable manner with the ML framework PyTorch (44). With PyTorch, PYSEQM takes advantage of modern graphics processing unit (GPU) hardware to greatly accelerate calculations while automatic differentiation enables the interface with a NN for the dynamic parameterization of the SEQM model. For these SEQM methods, the total energy is expressed as the sum of electronic energy *E_elec_* and pairwise nuclear Coulomb interaction energy *E_nuc_*:

[1] $$E_{\mathrm{tot}}=E_{\mathrm{elec}}+\sum_{i<j}E_{\mathrm{nuc},ij}+E_{D3H4}$$

[2] $$E_{\mathrm{elec}}=\text{Tr}[D(h+H)]/2$$

[3] H(D)=h+G(D),

where $E_{\mathrm{nuc},ij}$ is the nuclear interaction between atom *i* and *j*, and $E_{D3H4}$ is the energy correction on hydrogen bonding and dispersion from Rezac and Hobza (42). Per Eq. 2, the electronic energy *E_elec_* is obtained from single-particle density matrix ***D***, one-electron core Hamiltonian ***h***, and the entire Hamiltonian (Fock matrix) ***H*** given by Eq. 3. ***G*** is the Coulomb matrix, which depends on ***D*** and electron–electron interaction terms (*SI Appendix*, section S2). The PM3 model utilizes a minimal basis set containing only effective valence orbitals and truncates electron–electron interaction terms. As such, there is a limited number of empirical parameters that are used to construct ***h***, ***G***, and $E_{\mathrm{nuc},ij}$. With these empirical parameters replaced by the dynamic values learned from local atomic environments by the HIPNN network, the SEQM module first computes the core Hamiltonian ***h*** and Coulomb integrals ***G*** and then performs standard SCF procedure to get the density matrix ***D*** and entire Hamiltonian ***H***, as shown in Fig. 1*B*. Finally, the electronic energy *E_elec_* from Eq. 2, along with nuclear energies *E_nuc_* and the correction $E_{D3H4}$, is computed to get total energy, *E_tot_*. The gradient of total energy with respect to the molecular coordinates is automatically computed in PyTorch to get the atomic forces. Several issues from the SCF procedure along with the implementation and training details are referred to in *Methods*. We also train a pure NN model HIPNN and reoptimize the PM3 parameter set to create PM3*, as detailed in *Methods*.

## Results

### Learned Hamiltonian Parameters.

**Fig. 2.**
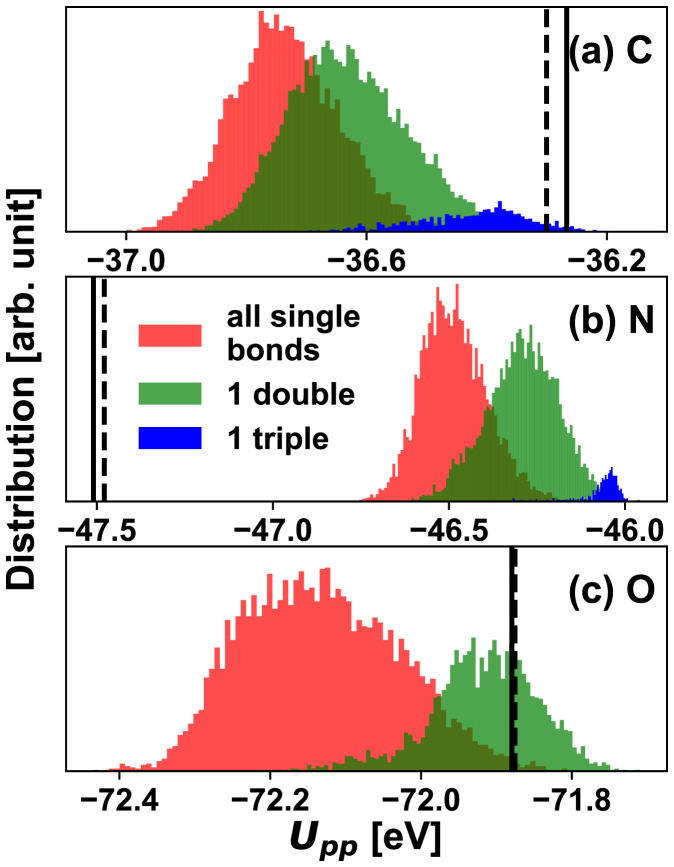
Histograms for the SEQM predicted parameter *U_pp_* (*p* orbital on site energy) from the Hamiltonian-based HIPNN+SEQM model for (*A*) C, (*B*) N, and (*C*) O. Histograms are colored based on atomic hybridization. The vertical lines are the constant PM3 (solid) and PM3* (dashed) values for each parameter.

We first examine the SEQM parameters in PM3* and the original PM3 models. As shown in Fig. 2 and *SI Appendix*, Table S2, the Hamiltonian parameters in the PM3* set have small deviations from the original PM3 values. Most deviations are within 10%, with few exceptions for several relatively small nuclear interaction parameters *K*_1_ and *K*_2_ of carbon atoms in *SI Appendix*, Table S2. As the dataset and optimization procedure used to generate the original PM3 parameter set are different from the ones used to create the PM3* parameter set (*Methods*), this difference is expected. In fact, the magnitude of the parameter shifts suggests the PM3 Hamiltonian is consistent with DFT quantum mechanics. These small differences also indicate that the performance improvement of PM3* over PM3 may be insignificant. Using the PM3* parameters as a starting point, the HIPNN module in the HIPNN+SEQM model learns to tune these parameters based on the local atomic environment. We apply the HIPNN+SEQM model on 8,000 randomly selected molecules (100,000 atoms) in the training set and extract the intermediate SEQM parameters and compare with the PM3 and PM3* geometry as well as molecule-independent parameters.

Among the nine output SEQM parameters for each element, most of them are similar to the corresponding PM3 parameters, as shown in *SI Appendix*, Fig. S1. We analyze in detail and exemplify trends using the on-site energy term *U_pp_* for the *p* orbital in the effective Hamiltonian. The distributions of most output parameters (such as *U_ss_* for the *s* orbital for H) are Gaussian-like (*SI Appendix*, Fig. S1), but some are asymmetric and non-Gaussian, showing multimodal behavior (Fig. 2). This is due to the complex chemical environments present in the training set. These multimodal distributions can be related to the local bonding environment or related to the electron hybridization in C, N, and O as shown in Fig. 2 *A*–*C*. For atoms with different order of bonding with surrounding atoms, their values of *U_pp_* show the following order: triple bond (sp hybridization) > double bond (sp ^2^ hybridization) > all single bonds (sp^3^ hybridization). The trend clearly shows that HIPNN+SEQM learned that hybridization should modify on-site orbital energies and illustrates an advantage of the dynamic parameterizations produced by the HIPNN+SEQM model.

### Model Performance.

The detailed performance breakdown of these models is listed in Table 1. On the held-out test set, PM3* reduces the energy errors by about 25% compared to base PM3, which is likely due to the testing data originating from the same DFT method used to optimize PM3*. Rigid parameterization is a limiting factor in PM3* since these errors are further decreased by more than 70% in the HIPNN+SEQM model. HIPNN has the best performance on the held-out test set with an energy mean absolute error (MAE) of 0.063 eV (1.45 kcal/mol) or 0.0049 eV per atom (0.15 kcal/mol per atom), which is in line with other results using ANI-1x datasets and pure NN models (48). However, this performance does not transfer to different and larger systems, as shown in the following sections.

**Table 1.** Accuracy on predicting atomization energies per atom (unit: eV) and atomic forces (unit: eV/Å) on the COMP6 subsets for the original PM3 model, the reoptimized PM3* model, the HIPNN model, and the Hamiltonian-based HIPNN+SEQM model

	Atomization energy/atom, eV				Atomic forces, eV/Å			
	PM3	PM3*	HIPNN	HIPNN+SEQM	PM3	PM3*	HIPNN	HIPNN+SEQM
Held-out test RMSE	0.046	0.032	**0.0073**	0.013	0.79	0.56	**0.20**	0.27
Held-out test MAE	0.033	0.024	**0.0049**	0.010	0.50	0.37	**0.10**	0.17
GDB RMSE	0.028	0.019	0.017	**0.009**	0.64	0.43	0.30	**0.22**
GDB MAE	0.022	0.014	0.013	**0.007**	0.42	0.28	0.19	**0.15**
S66x8 RMSE	0.045	0.035	0.045	**0.018**	0.48	0.34	0.43	**0.15**
S66x8 MAE	0.036	0.029	0.030	**0.014**	0.25	0.18	0.20	**0.10**
Drug Bank RMSE	0.023	0.031	0.046	**0.011**	0.54	0.37	0.43	**0.22**
Drug Bank MAE	0.018	0.027	0.038	**0.009**	0.35	0.25	0.27	**0.16**
Tripeptides RMSE	0.024	0.030	0.046	**0.008**	0.67	0.48	0.41	**0.26**
Tripeptides MAE	0.020	0.028	0.045	**0.007**	0.42	0.29	0.23	**0.17**
ANI-MD RMSE	0.030	0.034	0.076	**0.012**	0.56	0.35	0.66	**0.28**
ANI-MD MAE	0.026	0.032	0.055	**0.011**	0.37	0.25	0.38	**0.19**

Boldface indicates best performing model.

We next test all models to the COMP6 benchmark dataset to analyze their transferability to other molecular families and much larger systems. COMP6 is a sophisticated benchmark that includes various molecular conformers covering diverse organic and biochemical and conformational space. The subsets in COMP6 are two GDB (Chemical Universe Generated Database) subsets (GDB07to09 and GDB10to13) that are subsampled from GDB-11 (49, 50) and GDB-13 (51), respectively. The Drug Bank subset is a subsampling of the Drug Bank dataset (52). The tripeptide subset contains 248 random tripeptides. Diverse normal mode sampling (DNMS) (48) is used to create nonequilibrium conformations for these four subsets. The ANI-MD subset is generated from MD simulations using ANI-1x potential on 14 drug molecules (48). Finally, the S66 × 8 subset is created from the original S66 × 8 benchmark and contains 66 dimeric systems, which focuses on noncovalent interactions in biological molecules, including hydrogen bonding, *π*–*π* stacking, and van der Waals interactions (53).

The performance of all models on COMP6 is summarized in Table 1 and *SI Appendix*, Figs. S2 and S3. Overall, the HIPNN+SEQM model performs much better than both the pure NN and semiempirical models for predicting both molecular energies and atomic forces. As shown in Table 1, the HIPNN+SEQM model has a root-mean-square error (RMSE)/MAE more than 50% lower when compared to PM3*. On the S66 × 8 subset, HIPNN+SEQM shows again consistent accuracy when predicting total system energy and has smaller atomic force errors, partially due to the narrow range of noncovalent interactions. This is in contrast with the other three models, which all show a significant loss of accuracy when predicting energies for S66 × 8 and show skews when predicting atomic forces indicating systematic errors (*SI Appendix*, Figs. S2a-2–d-2 and S3 a-2–d-2). For the other COMP6 subsets, Drug Bank, Tripeptides, and ANI-MD, which contains much larger biological molecules up to 300 atoms, the Hamiltonian-based methods PM3, PM3*, and HIPNN+SEQM show consistent performance when compared to the results on the smaller GDB sets. This indicates that the Hamiltonian-based methods exhibit good extensibility: They can make accurate predictions on systems much larger than those found in their training sets. In contrast, HIPNN alone exhibits a significant accuracy drop on the larger datasets, with atomization energy per atom errors 6 to 9 times larger and force errors 1.5 to 2 times larger than those observed on the GDB dataset, indicative of poor extensibility.

**Fig. 3.**
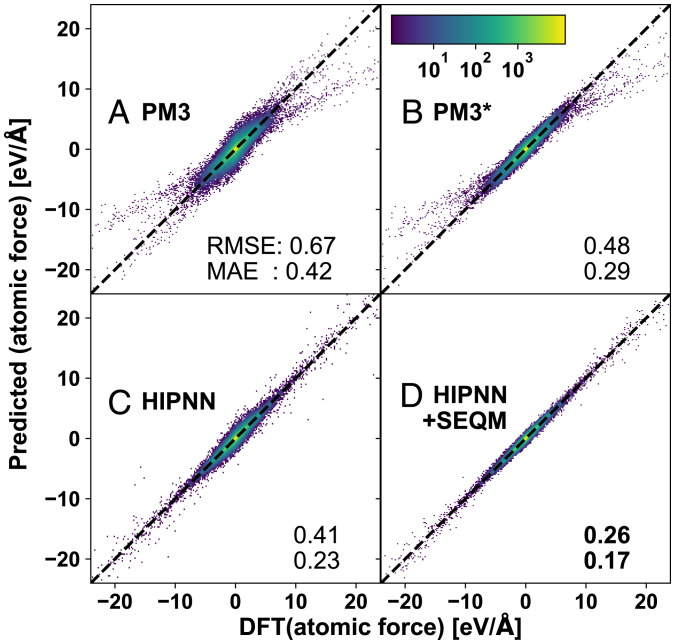
Two-dimensional histograms show predicted vs. DFT reference atomic forces on the COMP6 subset Tripeptides from four models in the main text: (*A*) original PM3, (*B*) reoptimizing PM3*, (*C*) pure HIPNN, and (*D*) Hamiltonian-based HIPNN+SEQM.

Compared to PM3 and PM3*, HIPNN+SEQM not only shows a significant improvement in accuracy, but also corrects a force skew observed in the tripeptide dataset (Fig. 3). Detailed examination shows these force outliers originate mostly from N-H bonds with lengths between 0.7 and 0.9 Å, which are much shorter than average N-H bonds with length 1.0 to 1.1 Å. Some other outliers come from C-C and C-H bonds as shown in *SI Appendix*, Fig. S4. The original PM3 parameter set is not fitted for this region, and while PM3* slightly corrects this discrepancy, the glaring problem remains (as illustrated in *SI Appendix*, Fig. S5). The failure of PM3* indicates there may be no static reduced parameterization capable of fitting potential energy surfaces simultaneously at near equilibrium and compressed geometries and that to capture all of these regions would require a reformulation of the semiempirical method. As a comparison, both HIPNN and HIPNN+SEQM models do not demonstrate this skew and show uniform improved performance for compressed, stretched, and normal bond lengths. Thus, by dynamically reparameterizing an established semiempirical model, we demonstrate that we can expand the region of phase space where applicable.

**Fig. 4.**
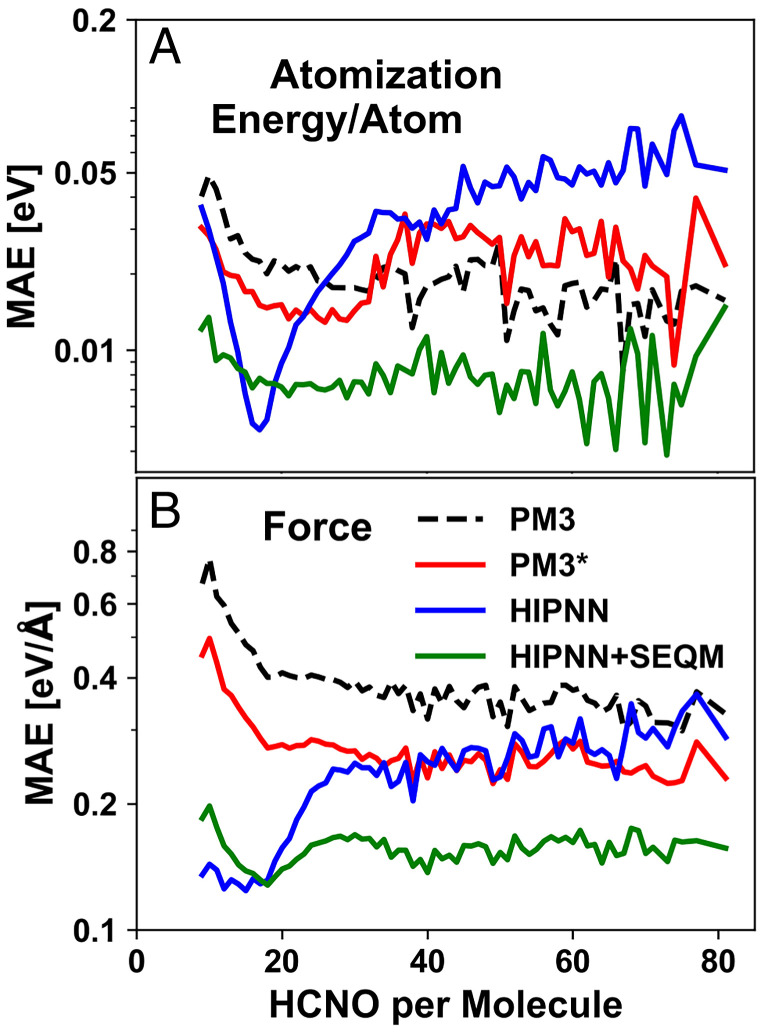
MAEs of (*A*) atomization energy/atom and (*B*) atomic forces as a function of a system size in COMP6 for the four models: PM3 (black dashed line), PM3* (red solid line), HIPNN (blue solid line), and HIPNN+SEQM (green solid line). To obtain acceptable statistical sampling of the MAE, we restrict this analysis to system sizes with more than 50 configurations.

The extensibility of the models is further examined in Fig. 4, which shows the average inference error with respect to the size of chemical systems in COMP6. To get a reliable estimation of errors with respect to system sizes, we compute the MAE for each system size with more than 50 configurations. As a general trend, the Hamiltonian-based models have relatively consistent errors with regard to system size, while the errors of the NN model increase with system size. This illustrates that models building upon quantum mechanical concepts capture the essence of predicting energies and forces, while the conventional heuristic NN model may require further augmentation such as including long-range corrections or training to larger molecules to achieve this level of accuracy. Both machine-learning methods (HIPNN and HIPNN+SEQM) have similarly small errors when applied to small systems with up to 20 atoms, but the Hamiltonian-based HIPNN+SEQM method maintains this performance for larger systems (Fig. 4).

### Predicting Properties of Optimized Structures.

To test whether HIPNN+SEQM can find the correct geometric minimum for these molecules, we optimize systems from the Drug Bank subset of COMP6 with each model and compute bonds, angles, dihedral angles, and the vibrational spectrum for each molecule. The results of this analysis, for every model type, are compared against the reference DFT calculations using the *ω* B97X functional and 6-31G* basis set with the ORCA software package (54). The DFT optimization is performed with a geometric convergence tolerance of 1.5 × 10^–3^ eV/Å (3 × 10^–5^ hartree/bohr) on gradients.

The other models use the L-BFGS-B optimizer in SciPy with a tolerance of 10^–3^ eV/Å (55). Of the 837 molecular systems in this dataset, HIPNN optimizes to unphysical structures for about 40% of the systems. These systems have been removed from the following comparison for the HIPNN model only, which artificially inflates the HIPNN performance scores. With the optimized structures, the RMSD* between these structures and the corresponding structures from DFT is calculated with the RDKit package (56) and Hessian matrices are diagonalized to obtain vibrational frequencies. As ORCA gives unphysical frequencies for 179 systems due to insufficient optimization, they are removed from this comparison. There are no such failures with PM3, PM3*, and HIPNN+SEQM for optimization and vibrational analysis, so all remaining structures are used to compute vibrational frequencies shown in Table 2. HIPNN, however, fails to give sufficiently optimized structures for 405 systems and yields unphysical frequencies, which are filtered from the HIPNN analysis, artificially improving its results. Dihedral angles for sp^3^ hybridized N atoms are also shown because PM3 is known to have problems with these quantities.

**Table 2.** Bond length, angle, dihedral angle, vibrational frequency, and RMSDs for PM3, PM3*, HIPNN, and HIPNN+SEQM models on 658 optimized structures from Drug Bank subset in COMP6

RMSE/MAE	PM3	PM3*	HIPNN	HIPNN+SEQM
Bond length, Å, RMSE	0.017	0.010	0.013	**0.006**
Bond length, Å, MAE	0.013	0.007	0.007	**0.004**
Bond angle, ${}^{{}^{\circ}}$, RMSE	2.08	2.20	2.62	**1.58**
Bond angle, ${}^{{}^{\circ}}$, MAE	1.42	1.41	1.46	**1.14**
Dihedral 1-35-5 angle, ^∘^, RMSE	**8.60**	9.06	12.76	9.14
Dihedral 1-35-5 angle, ${}^{{}^{\circ}}$, MAE	5.92	6.17	6.71	**5.44**
Frequency, cm–1, RMSE	78.2	83.1	168.0	**54.2**
Frequency, cm–1, MAE	58.9	65.8	47.8	**32.6**
Å mean ± SD, RMSD	0.69 ± 0.62	**0.68** ±**0.57**	0.96 ± 0.77	0.70 ± 0.52

Boldface indicates best performing model.

**Fig. 5.**
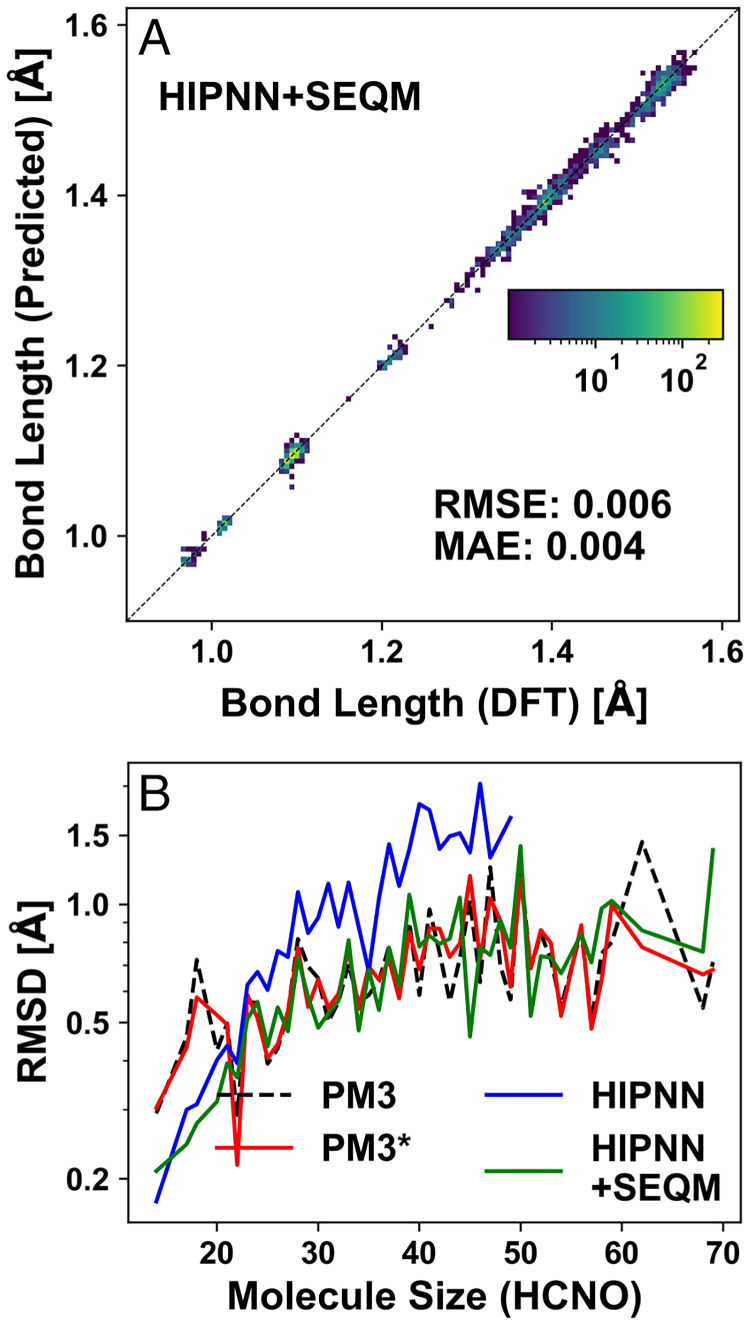
(*A*) Two-dimensional histogram showing predicted bond lengths vs. the DFT reference. (*B*) Dependence of RMSD on system size between optimized structures from PM3 (black dashed line), PM3* (red solid line), HIPNN (blue solid line), and HIPNN+SEQM (green solid line) compared to DFT-optimized geometries, where each value is an average of six or more systems with the same number of atoms.

As listed in Table 2, HIPNN+SEQM performs consistently better than other models in predicting local properties such as bond lengths and angles, dihedral angles, and vibrational frequencies. PM3 systematically underestimates O/N-H (around 1 Å) and overestimates C-H bond length (around 1.1 Å) by 0.02 Å as shown in *SI Appendix*, Fig. S7*A*. PM3* and HIPNN+SEQM correct this downshift as shown in *SI Appendix*, Fig. S7*D* and Fig. 5*A*. The accuracy for predicting bond angles is also slightly improved over PM3 for PM3* and HIPNN+SEQM. As shown in *SI Appendix*, Fig. S7 *B* and *E*, PM3 and PM3* display a Z-shape distribution on dihedral angles: small errors for dihedral angles around 0${}^{{}^{\circ}}$ (planar shape for sp^3^-hybridized N atom and its bonded atoms) and around 30 to 40${}^{{}^{\circ}}$ (pyramidal shape) and large errors for dihedral angles slightly away from 0${}^{{}^{\circ}}$ (5 to 20${}^{{}^{\circ}}$). It is known that PM3 failed to give planar amide bonds in peptides (57) and HIPNN+SEQM performs slightly worse than PM3 and PM3* here, as there is no Z shape as shown in *SI Appendix*, Fig. S7*B*.

HIPNN+SEQM also provides an improvement over PM3 and PM3* for vibrational frequency calculations over all ranges. There are roughly three ranges for frequencies in the selected organic systems as shown in *SI Appendix*, Fig. S6*A*: 3,300 cm^–1^ and above stem from O-H and N-H bonds, 2,900 to 3,300 cm^–1^ originate from C-H bonds, and lower than 2,000 cm^–1^ frequencies are attributed to other bond vibrations. PM3 and PM3* show a bias on frequencies over 1,500 cm^–1^ as shown in *SI Appendix*, Fig. S7*C* and *F*. This emphasizes that PM3 and PM3* methods give inaccurate energy curvatures for O/C/N-H bonds. However, after training, HIPNN+SEQM corrects these systematic errors, giving correct optimized distances as shown in Fig. 5*A* for these bonds, while slightly overestimating certain high frequencies.

Although HIPNN+SEQM shows a consistent improvement for predicting local properties, there is no improvement when predicting the optimized structures, as shown in Fig. 5*B* and the minimal root-mean-square displacement (RMSD) reported in Table 2. In Fig. 5*B*, HIPNN+SEQM performs nearly identically to PM3*, with slightly smaller RMSDs for small systems and slightly larger RMSDs for large systems. All semiempirical methods show similar dependence on system size. On the other hand, HIPNN shows a significant increase in RMSDs for systems with more than 30 atoms, even with 40% of the systems removed due to unphysical optimized structures.

### Performance for Nonequilibrium Configurations.

A major challenge for ML-derived force fields (i.e., interatomic potentials) is ensuring consistent performance when operating far from equilibrium configurations, like those encountered during high-temperature MD simulations. This is largely a training dataset generation problem since many methods for sampling rely on room temperature MD. As temperatures rise, the distributions of molecular properties may shift, leading to unseen atomic environments. Advanced sampling can help alleviate this problem, like sampling all possible normal modes in the configuration space as was done in in ANI-1x (16). Here we examine how temperature affects a variety of property predictions for our HIPNN+SEQM model.

**Fig. 6.**
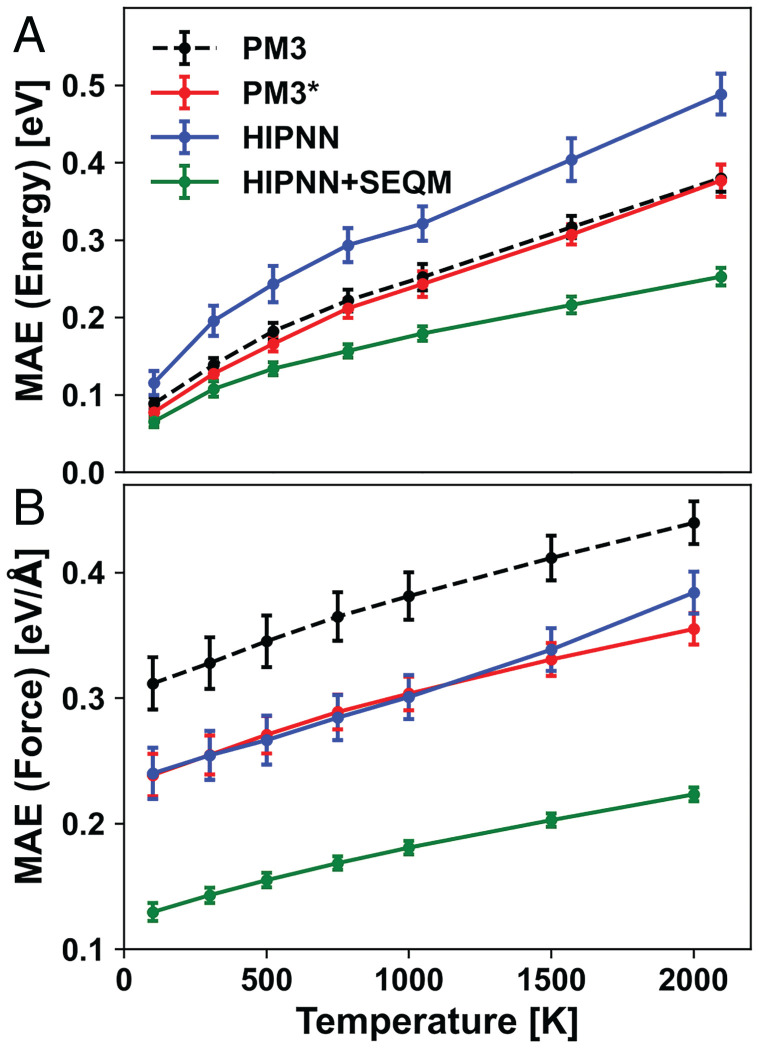
(*A*) Predicting errors on atomization energy over temperatures for PM3 (black dashed line), PM3* (red solid line), HIPNN (blue solid line), and HIPNN+SEQM (green solid line). For visual clarity, the error bar is shown as ±1/3 *σ* (SD). (*B*) Prediction errors of forces over temperatures for these four models. Same line styles are used with error bar shown as ±1/3 *σ*.

Forty-eight molecules in Drug Bank with 39 to 41 atoms (average 40 atoms) were heated to a specific temperature between 100 and 2,000 K with PM3 as implemented in PYSEQM. After 10 ps of heating, a 50-ps trajectory is generated for each system with an NVT ensemble (an ensemble that conserves number of particles, volume, and temperature) with a Langevin thermostat at the target temperature and a 1-fs time step. One configuration is extracted per 1 ps from each 50-ps trajectory, resulting in 2,400 configurations for each temperature. Potential energies and forces are computed for each configuration using all four models and reference DFT with the functional *ω* B97X and 6-31G* basis set. The inference errors of atomization energies and atomic forces are shown in Fig. 6.

As shown in *SI Appendix*, Fig. S8*A*, energy fluctuations show a linear dependence on system temperature, which is expected from the Boltzmann distribution of total energies in a system. The errors on predicting energies follow the same trend, increasing almost linearly for T > 500 K. The HIPNN+SEQM has the slowest error rate rise among the four methods, as illustrated in Fig. 6*A*. While the fluctuation of atomic forces shows a sublinear dependence on temperatures in *SI Appendix*, Fig. S8*B*, the errors on predicting forces increase linearly with temperature. Again, the HIPNN+SEQM increases the slowest of all methods (Fig. 6*B*). This indicates that HIPNN+SEQM has a wider range of geometries over which it can make reliable predictions compared to other semiempirical and ML methods and can be safely used for MD at higher temperatures.

### Transferability to HOMO-LUMO Prediction.

**Fig. 7.**
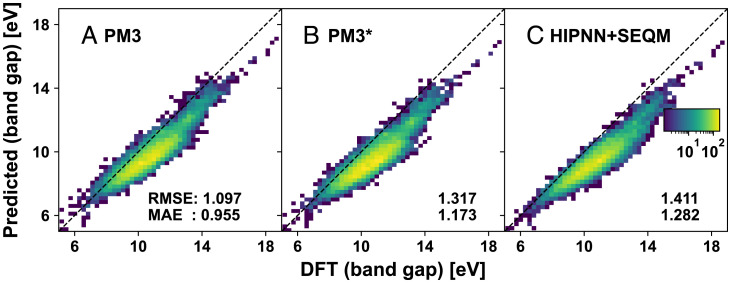
Two-dimensional histograms show the predicted band gaps from (*A*) PM3, (*B*) PM3*, and (*C*) HIPNN+SEQM models vs. DFT reference values.

To examine the performance on untrained properties of the Hamiltonian-based models, we compute HOMO-LUMO (Highest Occupied Molecular Orbital-Lowest Unoccupied Molecular Orbital) energy gaps for 10,000 molecular configurations randomly selected from the training dataset and compare them against values calculated with the same DFT settings. Comparisons between PM3, PM3*, and HIPNN+SEQM are shown in Fig. 7, while HIPNN, as a pure neural network, is unable to make predictions on properties to which it is not explicitly trained. Here, PM3* and HIPNN+SEQM slightly increase the errors on the HOMO-LUMO gap compared to PM3. Critically, while neither PM3* nor HIPNN+SEQM was explicitly trained to HOMO-LUMO gaps, their predictive performance is only slightly shifted. This shows the Hamiltonian-based model retains the structure and knowledge between the tasks of predicting unrelated quantities. This is usually a challenge for pure NN models that necessitates advanced techniques such as transfer learning (58). Additionally, the prediction of the HOMO-LUMO gap requires global information, namely the structure of the HOMO and LUMO orbitals, which makes it a particularly challenging quantity for traditional NN models. Our Hamiltonian-based framework resolves this by adapting the quantum structure into a machine-learning model that can automatically and simultaneously make accurate predictions for many types of observables.

## Discussion and Concluding Remarks

In summary, we demonstrate the performance improvement by implementing the quantum domain knowledge from semiempirical methods into the NN framework. We combine conventional neural net (HIPNN) with a semiempirical Hamiltonian (SEQM) module to produce a hybrid semiempirical model (HIPNN+SEQM) retaining essential quantum mechanical concepts. Much like previous work that attempted to devise empirical functional formulas for describing interactions in SEQM methods (59), HIPNN+SEQM allows the parameters that comprise the semiempirical Hamiltonian to change with a specific atom’s chemical environment. NNs, which are effectively general functional forms, appear to be ideal for finding these implicit, empirical functions. The incorporation of a NN to dynamically alter the SEQM parameters immediately improves the accuracy of the method by around 60% on predicting molecular energies and forces, while negligibly increasing the computational cost. It also corrects the bias errors of SEQM methods on predicting bond lengths and vibrational frequencies. At the same time, this Hamiltonian-based HIPNN+SEQM model shows much better extensibility compared to the pure NN architectures such as HIPNN when applied to systems much larger than those included in the original training set. While conventional NN-based models show unphysical performance for long-range interaction cases due to adapting to local features for near-sighted assumption, HIPNN+SEQM remains consistently accurate for large systems. Moreover, improved accuracy extends the usage of the model to higher-temperature regions that sample highly nonequilibrium configuration.

In addition, the environment-dependent Hamiltonian parameters also greatly alleviate the transferability problem of semiempirical methods that originates from the orthogonal compressed basis set (60). While it is problematic to describe systems with very different structures using constant Hamiltonian parameters in traditional SEQM, the NN-inferred Hamiltonian parameters can adapt to the change of local structure, adjusting their values accordingly, to improve the transferability. Besides excellent transferability, another important advantage for the physics-inspired HIPNN+SEQM approach is a smaller data requirement. The transferability and extensibility results shown here were achieved with only 61,842 training data points. This is 10 times less data than are used to train the HIPNN reference model and nearly two orders of magnitude less than the full ANI-1x dataset. This supports a different paradigm of ML models that incorporate physics to accurately simulate different classes of molecular systems. These ML models will also have drastically reduced training data requirements, limiting the amount of time spent running expensive ab initio calculations. Because the HIPNN+SEQM model relies on well-established quantum chemistry, it also opens more opportunities for interpreting the ML predictions. To emphasize that HIPNN+SEQM is indeed capturing the correct underlying physics, we reiterate that it performs only slightly worse than the original PM3 in predicting molecular HOMO-LUMO gaps, a property that was not used in the training procedure.

Although HIPNN+SEQM represents an improvement over the original PM3, some problems from SEQM models remain. It still performs poorly for cases involving torsion rotations and hydrogen bonding, inheriting some known drawbacks of SEQM methods (43, 61) (*SI Appendix*, section S3). We include the D3H4 correction to address this (42); however, the improvement on hydrogen bonding and proton affinity is minimal as shown in *SI Appendix*, Fig. S10 *D–I* and there is no improvement on torsion rotation (*SI Appendix*, Fig. S10 *A–C*). Long-range noncovalent bonding is strengthened, which gives slightly better energy barriers for hydrogen bonds. This can be attributed to the training dataset, which primarily consisted of small molecules and overemphasizes short-range interactions, leaving the HIPNN+SEQM parameters for long-range interactions fixed. This is demonstrated by HIPNN+SEQM’s success at predicting local properties like bonds, angles, and vibrational frequencies, while producing optimized structures with similar RMSDs. To address this issue, it may be possible to augment the training dataset with an active-learning approach (62) or utilize other sophisticated semiempirical methods like OMx (semiempirical models with orthogonalization) (45) and GFN2-xTB (semiempirical quantum approach providing multipole electrostatics and density-dependent dispersion contributions) (63) that can be interfaced with a NN in a similar way.

A very promising application for ML-optimized SEQM models is in the study of excited-state dynamics (64). Surface hopping and Ehrenfest dynamics for electronically excited molecules require many thousands of excited-state calculations, forcing them to use only inexpensive QM methods. It is reasonable to adapt HIPNN+SEQM or a similar blend of semiempirics with machine learning to increase the accuracy of the resultant excited states computed from a SEQM Hamiltonian. Here, we have shown that the HOMO-LUMO gap remains almost intact for the HIPNN+SEQM model compared to the parent SEQM models, suggesting that the wavefunction and Hamiltonian are suitable for excited-state calculations. Further improvements could be obtained by training the model explicitly to excited-state quantities. Another promising application of these methodologies is the study of chemical reactions, where electronic structure information, such as radical or charge states, can significantly change a molecule’s reactivity.

## Methods

As shown in Fig. 1, the HIPNN network learns the dynamical Hamiltonian parameters from the local atomic environments and passes them into the SEQM module. In PM3, for each element in the first three rows of the periodic table, there are 11 parameters for the electronic energy (4 for hydrogen), and 7 parameters for the nuclear energy. To prevent the overfitting of long-range interactions to the small-molecule systems, we train only a subset of these parameters. The entire set of PM3 parameters with brief description and the ones we train and generate with HIPNN are listed in *SI Appendix*, Table S1 and section S2. We apply the softplus function on the output of the inference layers for certain parameters, like the radial exponent for atom orbitals, as they are required to be positive based on their physical meaning or numerical constraints.

The density matrix D in Eq. **2** is obtained through the iterative SCF procedure leading to a mean-field description of the system, which stops when energy is converged to a predefined threshold (usually 10^–5^ hartree or tighter). This is usually achieved with specific algorithms like adaptive mixing, which we use here (43). The SCF algorithm can lead to three problems when interfaced with HIPNN-predicted parameters for training the model, which we discuss in *SI Appendix*, section S4. However, once the HIPNN+SEQM model is trained, all systems in all datasets discussed in this paper reach SCF convergence if proper care is given to SCF settings, such as mixing method, maximum number of iterations, and mixing rate of density matrix.

To accelerate the training, we start with a relatively loose convergence threshold of 10^–5^ *E_h_* (hartree) for the SCF procedure. The patience of the training procedure is 10 epochs, after which, if there was no improvement to the loss, the convergence threshold is decayed by a factor of 0.98 and the learning rate is decreased by a factor of 0.5. This continues until the convergence threshold reaches a minimum value of 10^–6^ *E_h_*. The total loss is defined as

[4] $$L=af(\hat{y}-y_{\mathrm{DFT}})*\sqrt{p}+bL_{L2}+cg(\Delta P).$$

Here *y* represents the target variables: In this work we include the total energies with bias removed and atomic forces. $L_{L2}$ is the L_2_ regularization term on the network and ΔP is the deviation of SEQM parameters. To constrain the predicted SEQM parameters and minimize the impact of SCF failures, we add the deviation of the SEQM parameters from the PM3* parameter set to the loss, which also behaves as a regularization from overfitting. We also scale the first term in the loss by $\sqrt{p}$, where *p* is the fraction of molecules in the batch whose SCF succeeded to converge (detailed in *SI Appendix*, section S4). *a*, *b*, and *c* are the weights for these loss components that are set to *a* = 1.0, *b* = 10^–6^, and *c* = 10, respectively, to minimize SCF failures and achieve stable training. *f* and *g* are error functions, for which we use a sum of RMSE and MAE for *f* and mean-square error (MSE) for *g*.

As the molecular configuration is used as the input for HIPNN to generate SEQM parameters, there is an implicit additional term present in the force calculation:

[5] $$\vec{F}=\frac{\partial E_{\mathrm{tot}}(\vec{R};P)}{\partial \vec{R}}+{\left(\frac{\partial P}{\partial \vec{R}}\right)}^{†}\frac{\partial E_{\mathrm{tot}}(\vec{R};P)}{\partial P}.$$

The first term is the normal one for computing force, and the second one is due to the change in parameterization predicted by HIPNN due to the changing atomic configuration and is somewhat akin to a Pulay force. With this full gradient, the total energy is fully conserved during MD simulations.

Hyperparameters and training the models closely follow the procedure detailed in ref. 8. Here we train both Hamiltonian-based (HIPNN+SEQM) and heuristic NN (HIPNN) models to compare the relative accuracy and extensibility for the two types of models. Both HIPNN+SEQM and pure HIPNN have two interaction layers, and each one has three consecutive on-site layers. In brief, there are 256 samples in each batch, and we use Adam (an optimizer derived from adaptive moment estimation) (65) with initial learning rate of 10^–3^ for training HIPNN and 10^–4^ for HIPNN+SEQM. Early stopping is used to terminate the training when 20 consecutive epochs without improvement are encountered. Additionally, using the same hyperparameters for gradient descent, we reoptimize the SEQM parameter set against the same dataset, resulting in the PM3* model, the parameter values of which are listed in *SI Appendix*, Table S2.

**Fig. 8.**
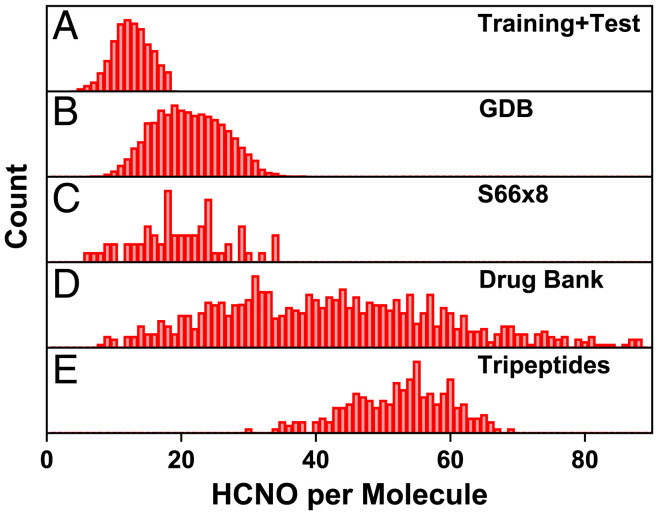
Distribution of molecule size used in training and testing. (*A*) The models are trained on a dataset with 5 to 18 H, C, N, and O atoms. (*B*–*E*) The models are tested on COMP6 datasets. ANI-MD is not depicted because it is composed of 14 molecules with 20 to 312 atoms.

We extract a subset of 618,409 samples containing all small conformers from the ANI-1x dataset (5 million samples), with 5 to 18 hydrogen, carbon, nitrogen, and oxygen atoms (1 to 13 C, N, O atoms), as shown in Fig. 8*A* (66). We also use the dataset’s reference quantities (energies and forces) computed at DFT level with the hybrid functional *ω* B97X and 6-31G* basis set using a Gaussian software package (67). The ANI-1x dataset contains nonequilibrium conformations generated with active learning to maximize the chemical and conformational diversity of data and to improve learning efficiency (16, 48). Due to the computational cost of training Hamiltonian-based models, only 10% of molecules (61,842 samples) are randomly chosen from this subset (8% for training, 1% for validation, and 1% for testing) for training HIPNN+SEQM and reoptimizing PM3*, while the entire subset is used to train HIPNN. We further compare the performance of the original PM3, reoptimized PM3*, pure HIPNN, and HIPNN+SEQM models on the COMP6 dataset, which consists of much larger molecules as shown in Fig. 8 *B*–*D*, with reference values generated with the same DFT settings. COMP6 contains six subdatasets: ANI-MD, Drug Bank, GDB07to09, GDB10to13, S66 × 8, and Tripeptides, randomly sampled from several sources with MD simulations as well as normal mode sampling to cover the broad organic and biochemical and conformational space (48). The molecule size difference can be further seen in *SI Appendix*, Fig. S9, which shows the pairwise atomic radial density. The training set and COMP6 have similar locations for first and second nearest-neighbor peaks, but the densities for these two datasets differ from 4 Å and above, indicating that a good cutoff to generate pair features for HIPNN is around 4 Å. Since the minimal pair distance is around 0.66 Å, we use a soft min distance of 0.65 Å and soft max distance 4.0 Å for the sensitivity functions used in HIPNN (8).

As the original PM3 model is fitted to heat of formation, and its basis set effectively includes only valence orbitals, the PM3 total molecular energy is very different from that obtained from DFT. As listed in *SI Appendix*, Table S3, due to the exclusion of core electrons, the absolute values of isolated atom energies for C, N, and O estimated with PM3 are an order of magnitude smaller than the values from DFT. As such, we first extract the energy biases for each type of atom through linear regression on the training dataset, as listed in *SI Appendix*, Table S3. All the reported results on energies regarding the PM3 model have removed these biases, as only the relative energy matters. To avoid the shift in the atomization energies, we train the model to the total energies with the bias removed in Eq. 1. We obtain the predicted atomization energies by adding back the bias and subtracting the isolated atom energy from DFT as listed in *SI Appendix*, Table S3.

## Supplementary Material

Supplementary File

## Data Availability

The code for HIPNN and the interface with PYSEQM has been deposited in GitHub at https://github.com/lanl/hippynn (68). The PYSEQM code and trained HIPNN+SEQM model can be found at https://github.com/lanl/pyseqm (69). The data used to train this model are available on Figshare (66). Any other data are available on request from the authors.

## Footnotes

*RMSD is the root mean squared error in atomic positions between two atomic structures where, one structure is translated and rotated freely to minimize the difference.
