# Supplementary material for: Deep learning of dynamically responsive chemical Hamiltonians with semiempirical quantum mechanics
Source: Proc Natl Acad Sci U S A. 2022 Jul 1;119(27):e2120333119. doi: 10.1073/pnas.2120333119 (PMC9271210; doi:10.1073/pnas.2120333119)
Supplement: Supplementary File [file pnas.2120333119.sapp.pdf]

PNAS
www.pnas.org

Supplementary Information for

Deep Learning of Dynamically Responsive Chemical Hamiltonians with Semi-Empirical Quantum Mechanics

Guoqing Zhou, Nicholas Lubbers, Kipton Barros, Sergei Tretiak and Benjamin Nebgen
Benjamin Nebgen
bnebgen@lanl.gov

This PDF file includes:

- Supplementary text
- Figures S1 to S10
- Tables S1 to S3
- SI References

Other supplementary materials for this manuscript include the following:

S1. HIPNN Architecture

The HIPNN (Hierarchically Interacting Particle Neural Network) architecture was detailed in Ref. (1). HIPNN models take molecular configurations as input, where each molecule is represented as a set of atom types and pairwise interatomic distances. This representation is invariant under translation, rotation, reflection, and permutations of the atomic coordinates. The input features are passed and shared through continuous message-passing layers as shown in Fig. 1(a) in main text. Within the HIPNN module, the atom *i* with atomic number Z_i in one molecule is represented using one-hot encoding as the initial input feature

$$z_{i,a}^{0} = \delta_{Z_i,\mathcal{L}(a)} \qquad [S1]$$

where $z_{i,a}^{0}$ is the a-th output feature from layer *l*=0 for atom *i*, (*l*, *i*, *a* are indexes for layers, atoms and features, respectively), $\mathcal{L}$ is the list of atomic numbers [1,6,7,8] for [H, C, N, O] for the mode reported in the main text. Features $z_{i,a}^{l}$ is then fed into the next layer *l*+1, which can be the on-site layers or interaction layers. The red blocks in Fig. 1(a) are on-site layers which are applied to the local features $\boldsymbol{z}_{i,a}^{\ell}$ for each individual atom as a fully connected neural network along with a residual network (2),

$$\tilde{\boldsymbol{z}}_{i,a}^{\ell+1} = f\left(\sum_b \mathbf{W}_{ab}^{\ell}\, \boldsymbol{z}_{i,b}^{\ell} + \mathbf{b}_a^{\ell}\right) \qquad [S2]$$

$$z_{i,a}^{\ell+1} = \sum_b \left(\widetilde{\mathbf{W}}_{ab}^{\ell} \tilde{\boldsymbol{z}}_{i,b}^{\ell+1} + \widetilde{\mathbf{M}}_{ab}^{\ell+1} z_{i,b}^{\ell}\right) + \tilde{\mathbf{b}}_a^{\ell} \qquad [S3]$$

where $\boldsymbol{W}_{ab}^{\ell}$, $\widetilde{\mathbf{W}}_{ab}^{\ell}$, $\widetilde{\mathbf{M}}_{ab}^{\ell+1}$ are learnable matrixes for the linear transformations acting on feature vector $\boldsymbol{z}_{i,b}^{\ell}$, $\tilde{\boldsymbol{z}}_{i,b}^{\ell+1}$, $z_{i,b}^{\ell}$, and $\mathbf{b}_a^{\ell}$, $\tilde{\mathbf{b}}_a^{\ell}$ are learnable biases for these linear transformations, *f*(x) is the nonlinear activation functions (here we use softplus function) and $\tilde{\boldsymbol{z}}_{i,a}^{\ell+1}$ is the output from activation functions and is fed into the residual layer in Eq. S3. The green blocks are interaction layers which pass information between nearby atoms, allowing atoms to see their chemical environment. The operation rule for interaction layers is

$$\tilde{\boldsymbol{z}}_{i,a}^{\ell+1} = f\left(\sum_{j,b}\left(\sum_{\nu} \mathbf{V}_{\nu,ab}^{\ell} \mathbf{s}_{\nu}^{\ell}(r_{ij})\right) \boldsymbol{z}_{j,b}^{\ell} + \sum_b \mathbf{W}_{ab}^{\ell}\, \boldsymbol{z}_{i,b}^{\ell} + \mathbf{b}_a^{\ell}\right) \qquad [S4]$$

where $\mathbf{s}_{\nu}^{\ell}(r_{ij})$ is the sensitivity functions with index ν defined in Ref. (1), the summation over atom index *j* collects information from neighboring atoms, $\mathbf{V}_{\nu,ab}^{\ell}$, $\mathbf{W}_{ab}^{\ell}$ and $\mathbf{b}_a^{\ell}$ are learned tensor, matrix, and bias for interaction layers, respectively. The object $\mathbf{V}_{\nu,ab}^{\ell}$ is the critical parameter tensor of HIPNN which provides for the structure of the interaction between different atoms; it characterizes how neighboring atoms will influence an atom based on their distances, which is selected via the sensitivity function $\mathbf{s}_{\nu}^{\ell}(r_{ij})$. All of the other matrices and biases in HIPNN play the standard role of fully connected neural network layer operating on each atom individually. A residual network is applied to get $z_{i,a}^{\ell+1}$ similarly as in Eq. S3. A sequence of on-site layers and interaction layers are used to process and share features. An inference layer is applied to the output

from each of the last on-site layers to obtain the output from the neural network in sequences to get zero- to higher order corrections of PM3 Hamiltonian parameters (3-5):

$$\boldsymbol{P}_i = \boldsymbol{P}_i^{PM3*} + \sum_n \boldsymbol{P}_i^n = \boldsymbol{P}_i^{PM3*} + \sum_n \left(\sum_a w_a^n z_{i,a}^n + \boldsymbol{B}^n \right) \qquad [S5]$$

Entry-wise additions are then used with the constant PM3 parameters (we use PM3* parameter set in this work) to yield the dynamic (local environment dependent) Hamiltonian parameters for the SEQM layers.

S2. SEQM methods and the parameters for training

We use the PM3 model from PYSEQM (6) in the main text. In PM3, slater-type atomic orbitals are used as basis functions:

$$\phi_{nlm} = R_{nl}(r) y_{lm}(\theta, \phi) \qquad [S6]$$

$$R_{nl}(r) = (2\zeta_{nl})^{n+1/2}[(2n)!]^{-1/2} r^{n-1} e^{-\zeta_{nl} r} \qquad [S7]$$

Here *n*, *l*, *m* are quantum numbers, y_{lm} is the real spherical harmonic function, ζ_{nl} is the orbital exponent.

The Hamiltonian ***H*** from PM3 method is constructed from one electron Hamiltonian ***h*** and Coulomb matrix ***G*** as follow:

$$\boldsymbol{H}(\mathbf{D}) = \boldsymbol{h} + \boldsymbol{G}(\boldsymbol{D}) \qquad [S8]$$

$$h_{\mu\nu} = \left\langle \mu \left| -\frac{1}{2}\nabla^2 \right| \nu \right\rangle - \sum_A Z_A \left\langle \mu \left| \frac{1}{R_A} \right| \nu \right\rangle \qquad [S9]$$

$$G_{\mu\nu} = \sum_{\lambda\sigma} D_{\lambda\sigma} \left[(\mu\nu, \lambda\sigma) - \frac{1}{2}(\mu\lambda, \nu\sigma) \right] \qquad [S10]$$

$$(\mu\nu, \lambda\sigma) = e^2 \iint \phi_\mu(\boldsymbol{r_1}) \phi_\nu(\boldsymbol{r_1}) \frac{1}{r_{12}} \phi_\lambda(\boldsymbol{r_2}) \phi_\sigma(\boldsymbol{r_2}) \mathrm{d}\boldsymbol{r_1} \mathrm{d}\boldsymbol{r_2} \qquad [S11]$$

Here Z_A is the effective charge for nuclei A with core shell electrons, $\mu, \nu, \lambda, \sigma$ are the valence shell atomic orbital index, $\frac{1}{R_A}$ is the one electron potential energy operator, and $-\frac{1}{2}\nabla^2$ is the kinetic energy operator. The density matrix $D_{\lambda\sigma}$ is computed from the molecular orbital coefficients of the occupied states:

$$D_{\lambda\sigma} = 2\sum_i C_{i\lambda} C_{i\sigma} \qquad [S12]$$

$(\mu\nu, \lambda\sigma)$ is the coulomb integral in chemistry convection as shown in Eq. S11. With the Neglect of Differential-Diatomic Overlap approximation applied in the SEQM methods in PYSEQM, ***h*** and is further simplified as:

$$h_{\mu\nu} = \begin{cases} U_{\mu\mu} - \sum_{B \neq A} Z_B(\mu\mu, s_B s_B) & \mu = \nu \\ -\sum_{B \neq A} Z_B(\mu\nu, s_B s_B) & \mu, \nu \text{ centered on atom } A \\ \beta_{\mu\nu} = \frac{(\beta_\mu + \beta_\nu)}{2} S_{\mu\nu} & \text{otherwise} \end{cases} \qquad [S13]$$

Here s is the s-orbital in the valence shell, β_μ/β_ν is the parameter for resonance integral, $S_{\mu\nu}$ is the overlap integral between orbital μ and ν. $U_{\mu\mu}$ is on-site orbital energy defined as:

$$U_{\mu\mu} = \langle \mu | -\nabla^2/2 | \mu \rangle - Z_A \langle \mu | 1/R_A | \mu \rangle \qquad [S14]$$

With NDDO, all three- and four-center integrals in ***G*** are ignored. The one center coulomb integrals in ***G*** are parameterized as:

$$(\mu_A \mu_A, \nu_A \nu_A) = g_{\mu\nu} \qquad [S15]$$

$$(\mu_A \nu_A, \mu_A \nu_A) = h_{\mu\nu} \qquad [S16]$$

Here $g_{\mu\nu}$ and $h_{\mu\nu}$ are the direct and exchange coulomb integrals between orbital μ and ν centered on atom A. They are reduced to parameters g_{ss}, g_{pp}, g_{p2}, g_{sp}, h_{sp}. Other one-center exchange and direct Coulomb integrals can be derived from these terms,

Additional parameters in PM3 are coming from the nuclear interaction part. In PM3, the nuclear interaction is described as:

$$E_{nuc}^{AB} = Z_A Z_B (s^A s^A, s^B s^B)[e^{-\alpha_A R_{AB}} + e^{-\alpha_B R_{AB}} + F_A(R_{AB}) + F_B(R_{AB})] \quad [S17]$$

$$F_A(R_{AB}) = \sum_{i=1}^{2} K_A^i \exp\left[L_A^i \left(R_{AB} - M_A^i\right)^2\right] \quad [S18]$$

Here α, K, L, M are parameters. All the parameters introduced above in PM3 are listed in Table SI below. There are in total 18 for C, N, and O atom, 11 for H as only s-orbital is used for hydrogen and there is no U_{pp}, ζ_p, β_p, g_{sp}, g_{pp}, g_{p2}, h_{sp} for H.

We statically optimize all the parameters listed in Table SI on the 10% of training dataset (61842 samples) and yield PM3*. For the Hamiltonian-based HIPNN+SEQM model, to avoid overfitting of long-range interaction from the training with small molecules, we restrict the model and only use HIPNN module to dynamically generates U_{ss}, U_{pp}, ζ_s, ζ_p, β_p, g_{sp}, g_{pp}, g_{p2}, h_{sp}. Here we exclude ζ_s, β_s, g_{ss} as we find the performance of computing vibrational spectra on large molecules are greatly improved if use constant values from PM3* for these 3. The parameters values in PM3 and PM3* are listed in Table SII. And the parameter distributions of U_{ss}, U_{pp}, ζ_s, ζ_p, β_p, g_{sp}, g_{pp}, g_{p2}, h_{sp} from HIPNN+SEQM are shown in Fig. S1.

S3. Cases where PM3 performs poorly

There are known cases where PM3 will give unreliable results, including the energy barrier of a torsion rotation, hydrogen bond energies and lengths, proton affinity, etc (7). D3H4 correction is used to address part of these issues and included in our PM3, PM3* and HIPNN+SEQM models. Here we apply the models to these cases, each with 3 systems where PM3 shows poor accuracy.

Torsion angles of H-C-C-H in Ethane, C-C=C-C in stilbene and C-N=N-C in azobenzene are examined. Structures are first generated through manual rotation of torsion angles in ethane and stilbene Nudged Elastic Band (NEB) (8) method for azobenzene. Constrained optimized is then performed using DFT with ωB97X/ 6-31G* to get energy profiles for these torsion rotations in Figs. S10 (a-c). For hydrogen bonding, 3 systems with weak, medium, and strong bonding are used: CH_4 -- NH_3, water dimer, and cyclic dimer of acetic acid. These system structures are first optimized with DFT, then we shift one molecule along the hydrogen bonding direction to get the energy profile with respect to the shift distance for all models, see Figs. S10 (d-f). To exam the hydrogen affinity, water, methanol and 4-hydroxy-2-pentanone are used. After geometry optimization with DFT, hydrogen atoms in the hydroxyl group of water and methanol are shifted along the bonding direction to get the energy profiles. In 4-hydroxy-2-pentanone, two structures with hydrogen bonding to different oxygen atoms are first optimized, and NEB calculation is followed to get the reaction path for the proton transfer, whose structures are used for energy profile calculation with all models, see Figs. S10 (g-i).

PM3 is notorious for poor torsional rotation barriers, especially those involving N atoms. In Fig. S6 (b), we see that HIPNN+SEQM slightly improves this performance on predicting sp^3 hybridized N torsional barriers. However, there is no obvious improvement on predicting energy barriers on torsion rotations for ethane, stilbene and azobenzene as shown in Figs. S10 (a-c). HIPNN+SEQM performs similarly to PM3 and PM3*, giving the wrong relative energy between trans- and cis-stilbene. Energy barriers are underestimated for all models in ethane and azobenzene. This indicates no improvement on torsion rotation after training on HIPNN+SEQM. However, the abysmal performance of HIPNN on these systems may point to a lack of data in the training set for torsional rotations. Specifically, in earlier ML force field work, torsions had to be specifically targeted when sampling in order to achieve reasonable performance (9, 10). Perhaps a similar approach would improve the performance of HIPNN+SEQM here.

For hydrogen bonding shown in Fig. S10 (d-f) (weak to median to strong strength in CH_4-NH_3, H_20 dimer, and cyclic dimer of acetic acid), PM3* performs as poorly as PM3 on the bond strength, and gives much smaller strengths than the values from DFT. HIPNN+SEQM improves on the bond strength, while underestimates the hydrogen bond length, 0.1 to 0.2 Å shorter than from DFT. The energy profile from HIPNN is unphysical and totally unreliable. We may do data augmentation to add hydrogen bonding in the training set to help alleviate the poor performance.

Last is to exam the energy changing from detaching proton in hydroxyl group in H_2O, CH_3OH and 4-hydroxy-2-pentanone in Fig. S10 (g-i). PM3+D3H4 and PM3*+D3H4 perform well on detaching proton from H_20 and CH_3OH, while give much higher barriers for the proton

transfer in 4-hydroxy-2-pentanone. HIPNN gives much lower energy barrier for proton transfer, but it is still considerably large. Moreover, HIPNN shows unphysical downshift of energy when detaching proton away from molecules. In contract, the HIPNN+SEQM model is overall better on these 3 cases, which much smaller errors on energy barriers from proton detaching and transfer.

Overall, there is no significant improvement of HIPNN+SEQM and PM3* model over PM3 on hydrogen bonding and torsion rotation where PM3 performs poorly, indicating cares must be taken on applying these models in these pitfalls, and shows future directions for improving the hybrid model. Further, pure NN model is totally not reliable here, suggesting poor transferability of pure NN model, and restricting the flexibility of applying NN based models.

S4. Challenges from Self-Consistent Field (SCF) procedure.

The density matrix $\boldsymbol{D}$ is obtained through the iterative SCF procedure leading to a mean-field description of the system. This is usually achieved with specific algorithms like adaptive mixing. The SCF algorithm leads to three problems when interfaced with HIPNN predicted parameters.

First, the SCF loop slows down the training of HIPNN not only on the forward pass, but also on the back-propagation pass. While the Hellmann-Feynman theorem allows the first order gradients of the total energy (or functions of total energy) to skip the SCF procedure in the backwards pass (11), this greatly limits the data available for model training, as many properties are not a function of total energy. In this work, we train our model explicitly to atomic forces, which requires the calculation of the second order gradients. Other example properties that could be targeted that would also require full backpropagation through the SCF procedure are dipoles, band gaps, polarizability, orbital energies, etc. As such, full backpropagation through the SCF procedure is enabled when the training target is not limited to total energy terms. The second challenge comes from memory usage associated with storing many intermediary matrices for the SCF backpropagation, which reduces achievable batch sizes by about an order of magnitude relative to a pure NN model. To address these two challenges, we use a small dataset and a relatively small batch size (described in the section: method in main text) to train the HIPNN+SEQM model.–Additionally, an upper bound of 50 SCF iterations was used reduce memory requirements.

The last and most serious problem is that the SCF may fail to converge to a true mean field limit or require too many steps to converge for certain molecules. This makes training unstable and leads to artificial loss variability, resulting in difficulty in updating the network parameters. Our chosen stochastic gradient descent algorithm, ADAM (12), evaluates the consistency with which a parameter is moved in a specific direction, and SEQM convergence failures can significantly hurt the training procedure. This difficulty is most pronounced when HIPNN outputs SEQM parameters that are very different from the original SEQM parameter set. To correct this, we apply a loss penalty (soft constraint) on the difference between the predicted HIPNN parameters and the re-optimized PM3* parameter set, as shown in Eq. 5 in main text, which is physically justified by the expectation that the parameters should form a sharp distribution due to varying chemical environment. Further, molecules that fail to converge after a pre-defined number of SCF iterations are screened from the first term in the loss function (see Eq. 4 in the main text) for that batch as the non-converged prediction is not reliable and unphysical, but included in the penalty loss function (third term in Eq. 4 in the main text). To reduce the variability in the loss, we also scale the corresponding loss (first term in following Eq. 4 in main text) by $\sqrt{p}$, where p is the fraction of molecules in the batch whose SCF succeed to converge. This is done with an assumption that the variance of gradients for each batch is inversely proportional to the effective batch size (13), or number of converged molecules samples here. In the work done by Yaron et al. with the DFTB TensorFlow layer for predicting atomic charges and dipoles (14), these problems from SCF procedure are avoided by disabling SCF loops with caching the charge density, and

updating the cache every few epochs. Though this can accelerate training to energy quantities, we observed in tests that it also amplifies the gradient noise related to atomic forces and reduces the stability of training between epochs. For this reason, we do not employ this caching strategy.

With the above practices applied SCF failure during training is minimized, only occurring with a few molecules in each batch of 256 systems. This has a minimal impact on training. During the testing or application phase, we can remove the upper bound constraint applied on the number of iterations for SCF procedure, along with switching the SCF algorithms, like constant mixing, adaptive mixing or Pulay implemented in PYSEQM (6). With the proper setting of SCF algorithms, there is no SCF failure when applying the trained model on the benchmark COMP6 dataset.

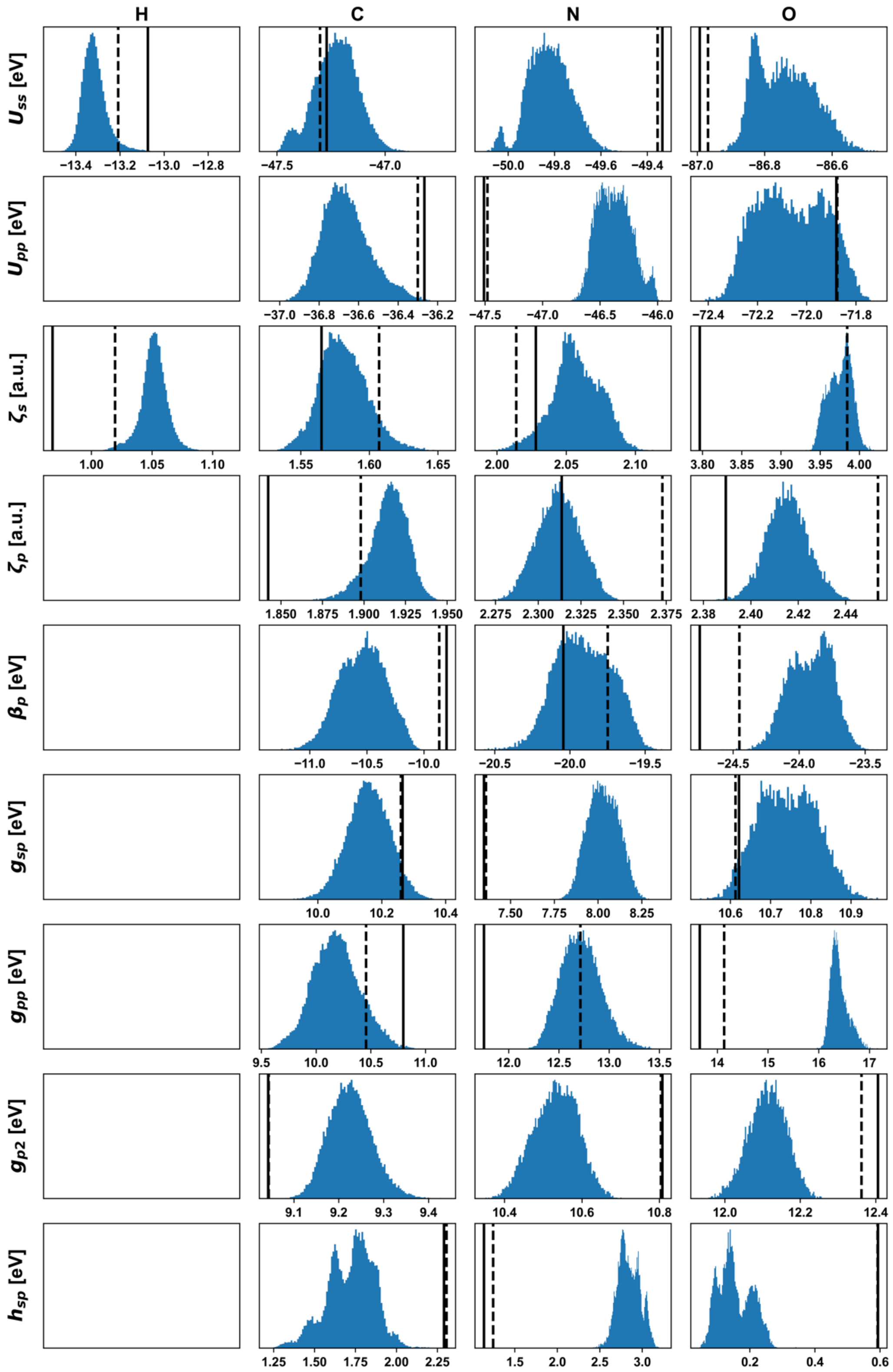

Fig. S1. Distributions [arbitrary unit] of parameters generated from HIPNN module in HIPNN+SEQM model, the solid and dash lines show the values from PM3 and PM3* parameter sets.

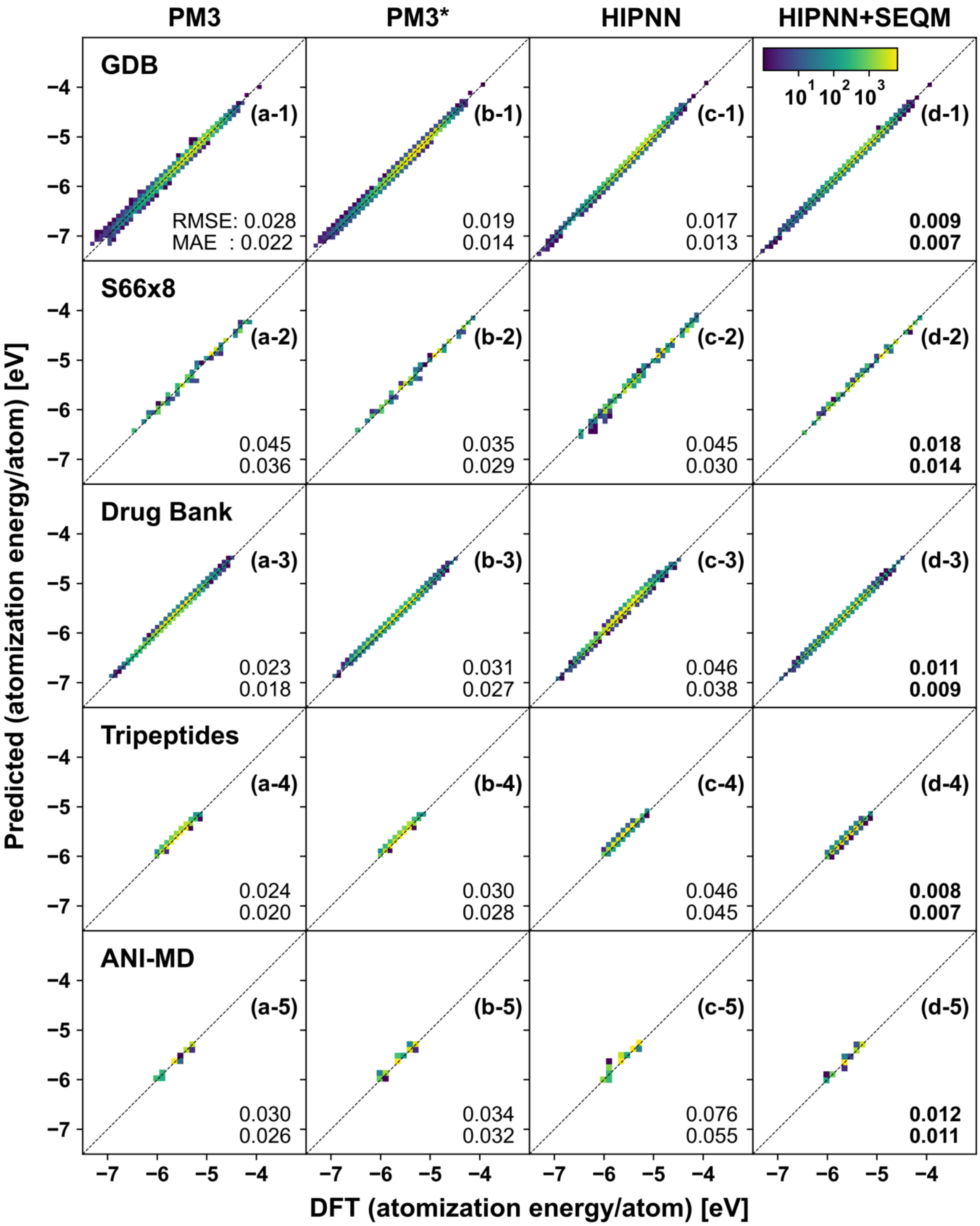

Fig. S2: 2-D histograms show predicted atomization energy/atom vs. the reference values from DFT calculations. The diagonal dash lines show the ideal case of a perfect agreement. From left to right, each column shows the results for PM3, PM3*, HIPNN and HIPNN+SEQM models. Each row shows the results on dataset GDB, S66X8, Tripeptides, Drug Bank and ANI-MD in COMP6. The values in the right bottom are RMSE and MAE for each model on each subset.

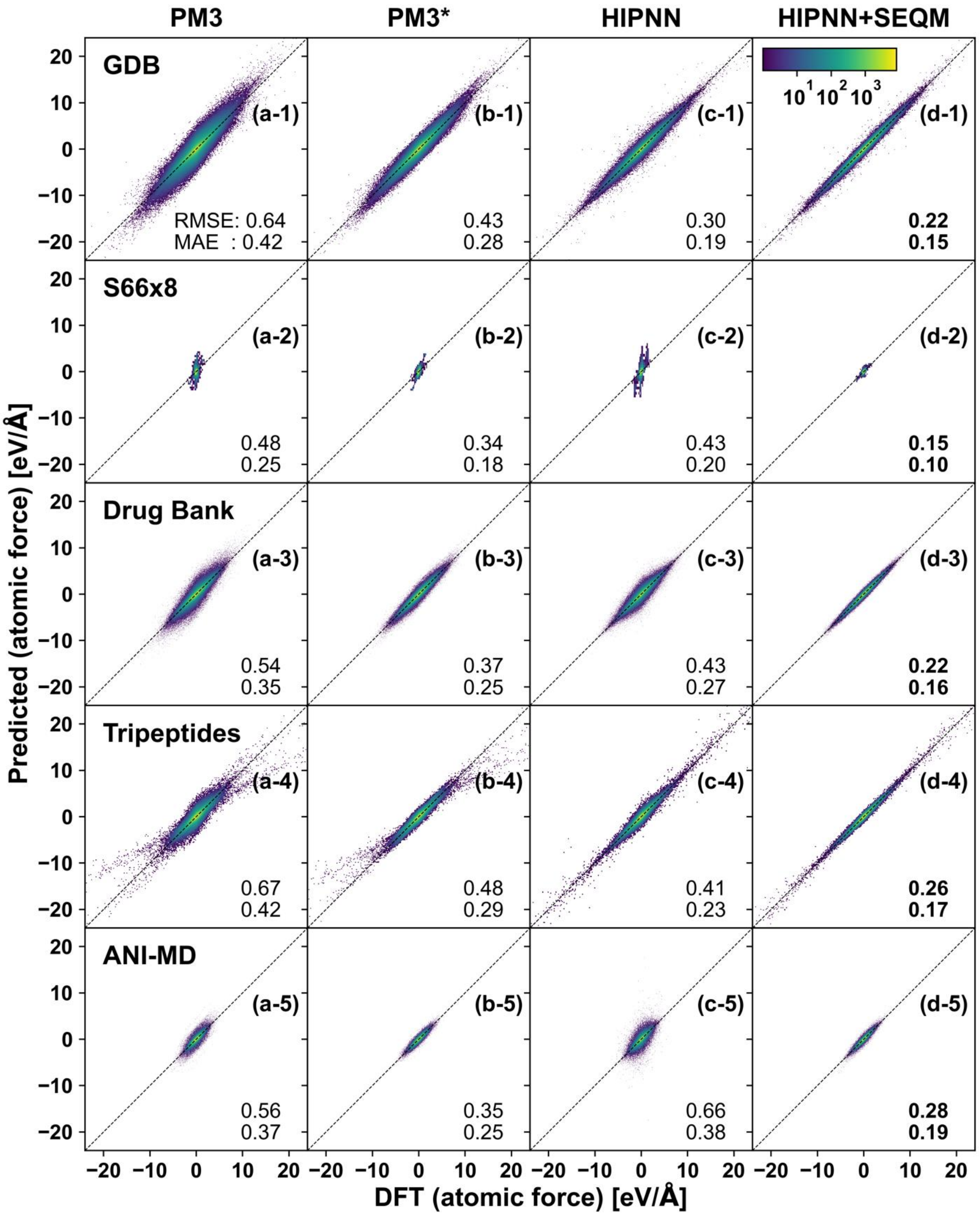

Fig. S3: 2-D histograms show predicted vs. DFT reference forces atomic forces. The diagonal dash lines show the ideal case of a perfect agreement. From left to right, each column shows the results for PM3, PM3*, HIPNN and HIPNN+SEQM models. Each row shows the results on dataset GDB,

S66X8, Tripeptides, Drug Bank and ANI-MD in COMP6. The values in the right bottom are RMSE and MAE for each model on each subset.

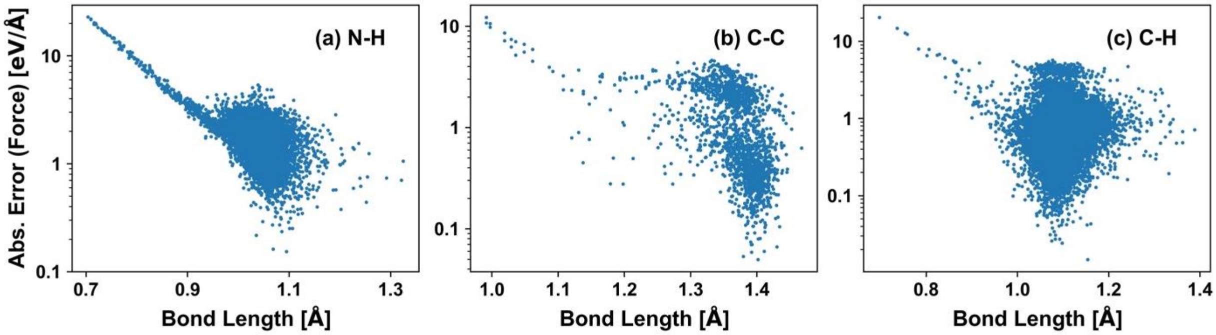

Fig. S4: Scatter plots of absolute error for atomic forces with respect to the bond length on the corresponding atoms for bonds (a) N-H, (b) C-C and (c) C-H on the original PM3 model testing on Tripeptides subset from COMP6.

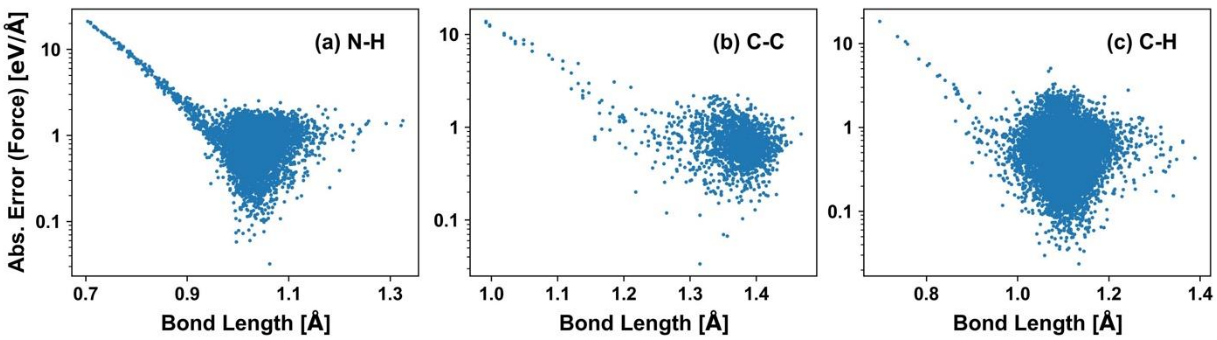

Fig. S5: Scatter plots of absolute error for atomic forces with respect to the bond length on the corresponding atoms for bonds (a) N-H, (b) C-C and (c) C-H on the re-optimizing PM3* model testing on Tripeptides subset from COMP6.

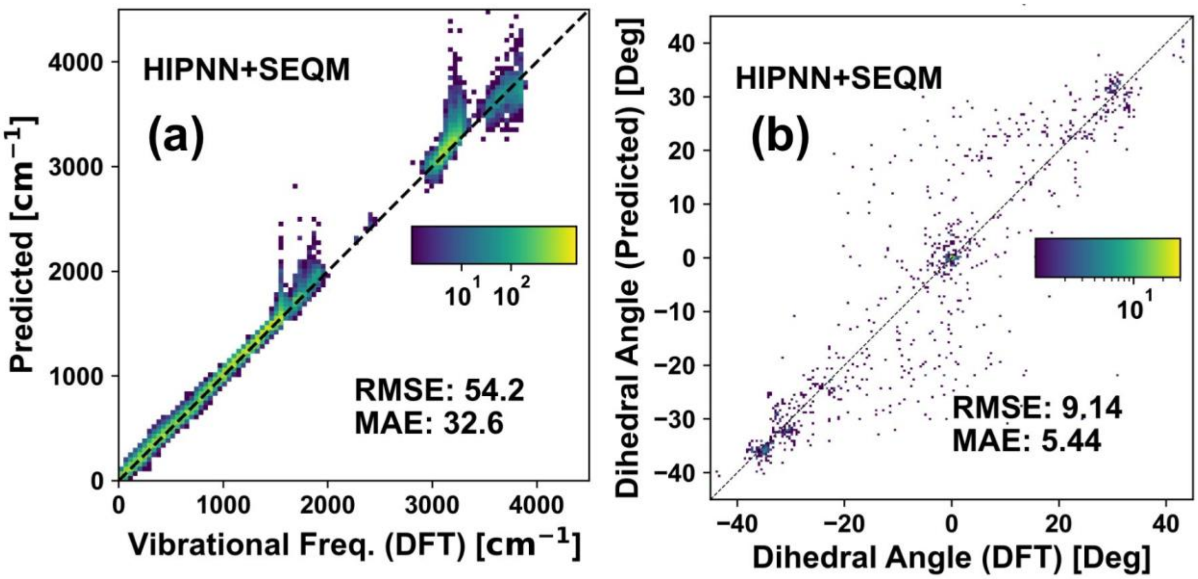

Fig. S6: 2-D histograms showing predicted (a) vibrational frequencies and (b) dihedral angles calculated with HIPNN+SEQM optimized structures in Drug Bank subset in COMP6.

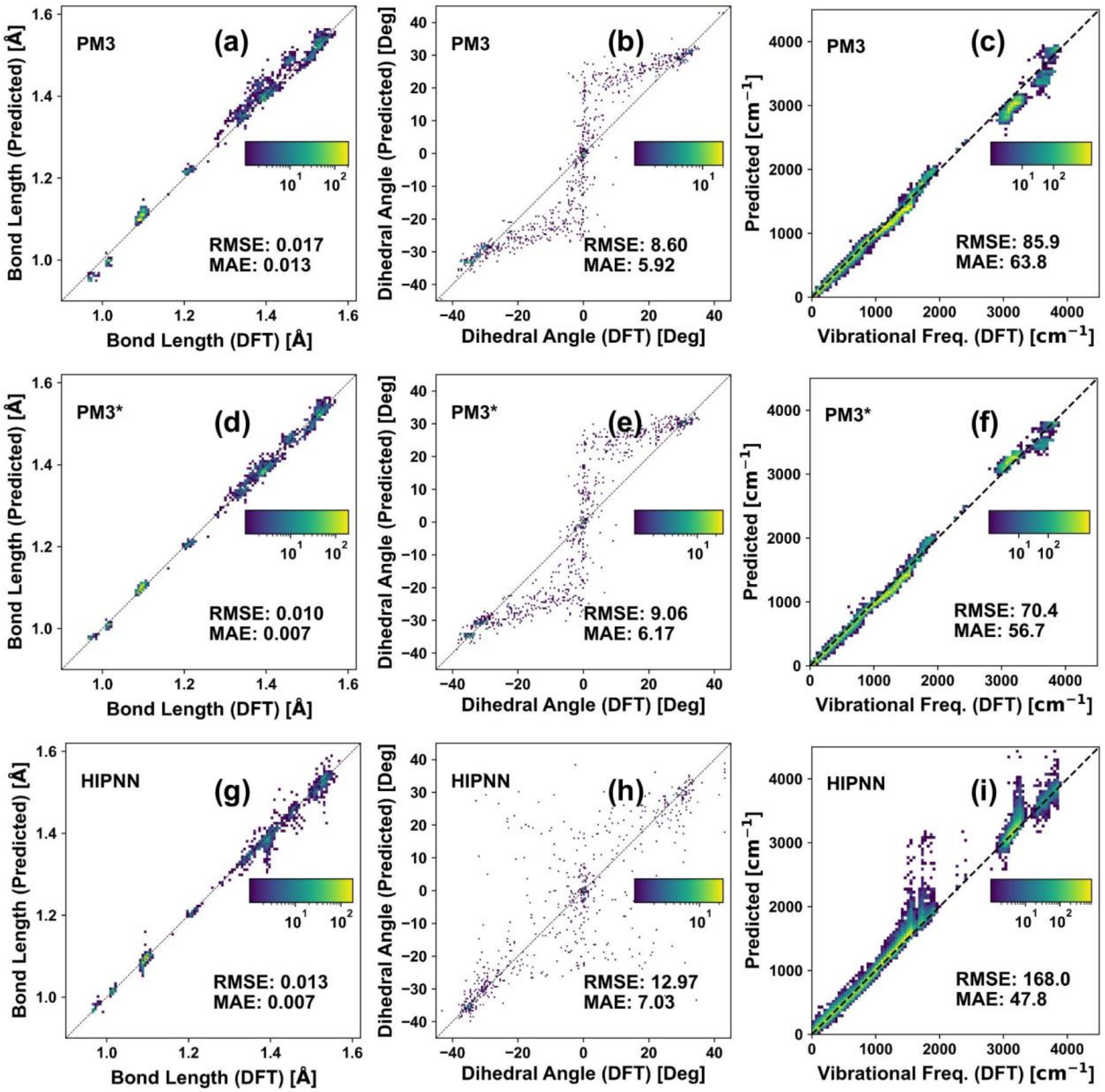

Fig. S7: 2D-histrograms show predicted bond lengths (a, d, g), dihedral angles for sp^3-hybridized N atoms (b, e, h) and vibrational frequencies (c, f, i) from PM3 (first row), PM3* (second row) and HIPNN (third row) vs. DFT reference values with the optimized structures in COMP6 subset Drug Bank with the corresponding model. Here for HIPNN, all the unphysical structures are removed in (g) and (h), and all the structures give unphysical frequencies are removed in (i), which makes the results looks much better than it is.

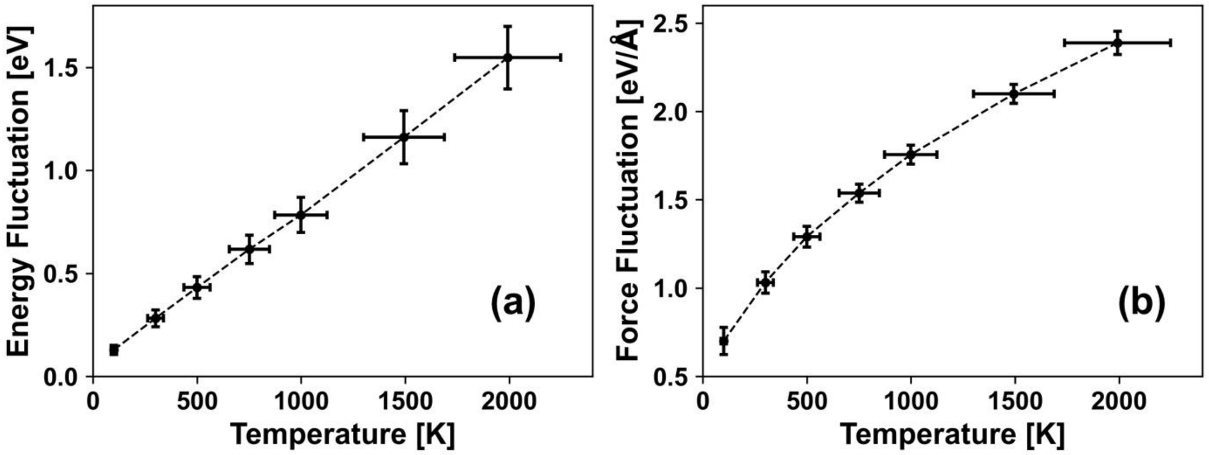

Fig. S8: (a) Fluctuation of total energy from DFT versus system temperatures from the molecular dynamic simulations of 48 molecules in Drug Bank (39 to 41 atoms), which is done with NVT ensemble using a Langevin thermostat. Deviation of fluctuation is estimated across 48 molecules. Deviation of temperature is estimated from the temperature of snapshots from the trajectories from the molecular dynamic simulations. (b) Fluctuation of atomic forces versus temperature, force deviation is estimated in the same way as energy.

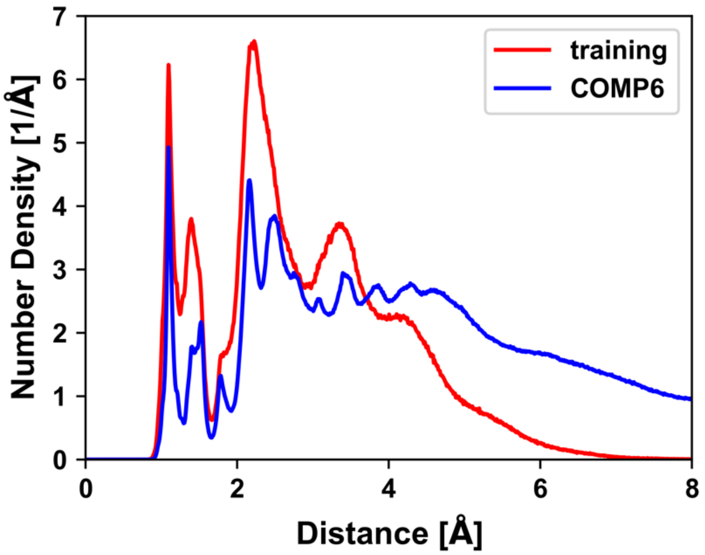

Fig. S9: Atomic radial density in the training dataset (red) and the COMP6 (blue) dataset.

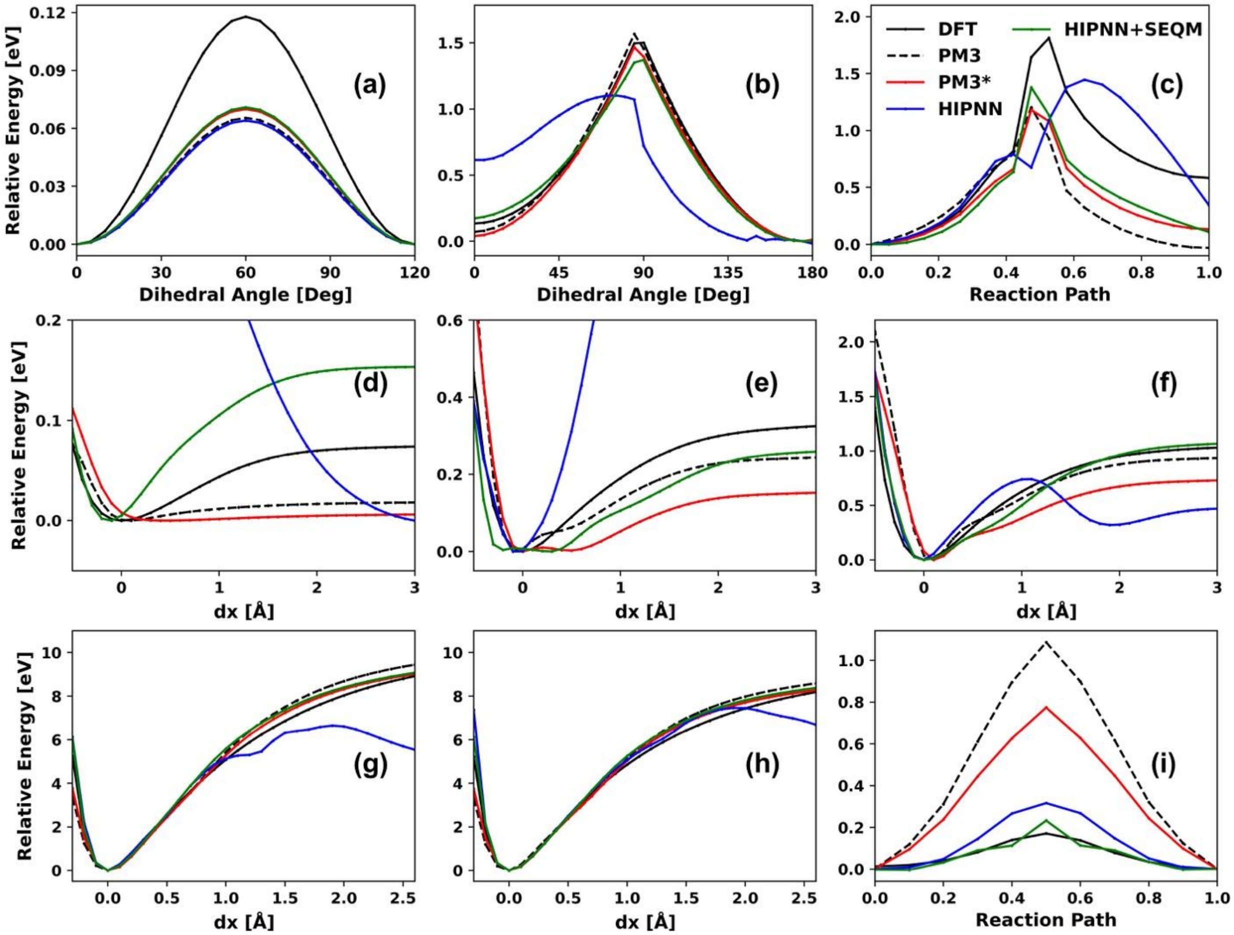

Fig. S10: Energy profiles on dihedral angles (a) H-C-C-H on ethane, (b) C-C=C-C in stilbene from trans (0°) to cis (180°), (c) C-N=N-C in azobenzene from cis to trans. Energy profiles on hydrogen bond length (relative to equilibrate length) on (d) CH_4 -- NH_3, (e) H_2O -- H_2O and (f) cyclic dimer of acetic acid. Energy profiles on detaching the proton in the hydroxyl group in (g) H_2O, (h) methanol and switching proton between hydroxyl and carbonyl group in (i) 4-hydroxy-2-pentanone.

Table S1: PM3 parameters with description.

SEQM parameters	Physical Meaning
U_{ss}	On-site energy for s-orbital, see Eq. S14
U_{pp}	On-site energy for p-orbital, see Eq. S14
ζ_s	radial exponent term for s-orbital, see Eq. S7
ζ_p	radial exponent term for p-orbital, see Eq. S7
β_s	Resonance parameter for s-orbital, see Eq. S13
β_p	Resonance parameter for s-orbital, see Eq. S13
g_{ss}	Direct coulomb integral term (ss, ss) for s-orbital centered on one atom, see Eq. S15
g_{sp}	Direct coulomb integral term (ss, pp) for s- and p-orbitals centered on one atom, see Eq. S15
g_{pp}	Direct coulomb integral term (p_ip_i, p_jp_j), (i, j=x,y,z, i ≠j) for p-orbitals on one atom, see Eq. S15
g_{p2}	Direct coulomb integral term (p_ip_i, p_ip_i), (i=j) for p-orbitals on one atom, see Eq. S15
h_{sp}	Exchange coulomb integral term (sp, sp) for s- and p-orbital on one atom, see S16
α	Exponent term in nuclear interaction, see Eq. S17
K_1, K_2	Weight terms for Gaussian terms in nuclear interaction, see Eq. S18
L_1, L_2	Scaling terms for Gaussian terms in nuclear interaction, see Eq. S18
M_1, M_2	Center terms for Gaussian terms in nuclear interaction, see Eq. S18

Table S2: Parameter values for original PM3 and re-optimizing PM3*

SEQM parameters	PM3				PM3*			
	H	C	N	O	H	C	N	O
U_{ss} [eV]	-13.073	-47.270	-49.336	-86.993	-13.208	-47.301	-49.356	-86.969
U_{pp} [eV]	-	-36.267	-47.510	-71.880	-	-36.3	-47.479	-71.875
ζ_s [a.u.]	0.968	1.565	2.028	3.797	1.019	1.607	2.014	3.984
ζ_p [a.u.]	-	1.842	2.314	2.389	-	1.898	2.373	2.454
β_s [eV]	-5.627	-11.910	-14.063	-45.203	-5.637	-12.019	-13.883	-45.017
β_p [eV]	-	-9.803	-20.044	-24.753	-	-9.866	-19.749	-24.453
g_{ss} [eV]	14.794	11.201	11.905	15.756	14.662	11.003	11.913	15.628
g_{sp} [eV]	-	10.265	7.349	10.621	-	10.26	7.36	10.612
g_{pp} [eV]	-	10.796	11.755	13.654	-	10.457	12.714	14.135
g_{p2} [eV]	-	9.043	10.807	12.406	-	9.044	10.804	12.362
h_{sp} [eV]	-	2.291	1.137	0.594	-	2.306	1.244	0.593
α [a.u.]	3.356	2.708	2.831	3.217	3.201	2.707	2.842	3.275
K_1 [a.u.]	1.129	0.050	1.502	-1.131	1.115	0.03	1.498	-1.136
K_2 [a.u.]	-1.060	0.051	-1.506	1.138	-1.059	0.038	-1.507	1.13
L_1 [a.u.]	5.096	6.003	5.901	6.002	5.173	7.007	5.957	5.971
L_2 [a.u.]	6.004	6.003	6.005	5.951	5.898	6.238	5.969	5.963
M_1 [a.u.]	1.537	1.642	1.711	1.607	1.6	1.558	1.718	1.607
M_2 [a.u.]	1.570	0.892	1.716	1.598	1.622	0.764	1.723	1.601

Table S3: Isolated atom energies for H, C, N, O from PM3 method and DFT. The biases of energies are evaluated with linear regression on the training dataset.

Isolated Atom Energy (eV)	H	C	N	O
PM3+D3H4	-13.073	-111.230	-157.614	-289.342
DFT	-13.587	-1027.614	-1482.010	-2039.222
Bias (eV) (PM3+D3H4 – DFT)	H	C	N	O
Total Energy	0.723	918.399	1327.500	1752.639
Atomization Energy	0.209	2.015	3.104	2.758

Uncategorized References

1. N. Lubbers, J. S. Smith, K. Barros, Hierarchical modeling of molecular energies using a deep neural network. *The Journal of chemical physics* **148**, 241715 (2018).
2. K. He, X. Zhang, S. Ren, J. Sun (2016) Deep residual learning for image recognition. in *Proceedings of the IEEE conference on computer vision and pattern recognition*, pp 770-778.
3. A. E. Sifain *et al.*, Discovering a transferable charge assignment model using machine learning. *The journal of physical chemistry letters* **9**, 4495-4501 (2018).
4. B. Nebgen *et al.*, Transferable dynamic molecular charge assignment using deep neural networks. *J. Chem. Theory Comput.* **14**, 4687-4698 (2018).
5. S. Magedov, C. Koh, W. Malone, N. Lubbers, B. Nebgen, Bond order predictions using deep neural networks. *J. Appl. Phys.* **129**, 064701 (2021).
6. G. Zhou *et al.*, Graphics processing unit-accelerated semiempirical Born Oppenheimer molecular dynamics using PyTorch. *J. Chem. Theory Comput.* **16**, 4951-4962 (2020).
7. O. Ludwig, H. Schinke, W. Brandt (1996) Reparametrisation of force constants in MOPAC 6.0/7.0 for better description of the activation barrier of peptide bond rotations. in *Molecular modeling annual* (Springer), pp 341-350.
8. H. Jónsson, G. Mills, K. W. Jacobsen, Nudged elastic band method for finding minimum energy paths of transitions. (1998).
9. J. S. Smith *et al.*, The ANI-1ccx and ANI-1x data sets, coupled-cluster and density functional theory properties for molecules. *Scientific data* **7**, 1-10 (2020).
10. J. S. Smith, B. Nebgen, N. Lubbers, O. Isayev, A. E. Roitberg, Less is more: Sampling chemical space with active learning. *The Journal of chemical physics* **148**, 241733 (2018).
11. R. P. Feynman, Forces in Molecules. *Phys. Rev.* **56**, 340-343 (1939).
12. D. P. Kingma, J. Ba, Adam: A method for stochastic optimization. *arXiv preprint arXiv:1412.6980* (2014).
13. X. Qian, D. Klabjan, The impact of the mini-batch size on the variance of gradients in stochastic gradient descent. *arXiv preprint arXiv:2004.13146* (2020).
14. H. Li, C. Collins, M. Tanha, G. J. Gordon, D. J. Yaron, A density functional tight binding layer for deep learning of chemical Hamiltonians. *J. Chem. Theory Comput.* **14**, 5764-5776 (2018).
